# Mathematical Modeling of Cell Death and Survival: Toward an Integrated Computational Framework for Multi-Decision Regulatory Dynamics

**DOI:** 10.3390/cells14221792

**Published:** 2025-11-14

**Authors:** Elena Kutumova, Ilya Akberdin, Inna Lavrik, Fedor Kolpakov

**Affiliations:** 1 Department of Computational Biology, Sirius University of Science and Technology, 354340 Sirius, Russia; 2 Laboratory of Bioinformatics, Federal Research Center for Information and Computational Technologies, 630090 Novosibirsk, Russia; 3 Translational Inflammation Research, Medical Faculty, Otto von Guericke University Magdeburg, 39106 Magdeburg, Germany

**Keywords:** apoptosis, autophagy, ferroptosis, immunogenic cell death, necroptosis, pyroptosis, ODE-based modeling

## Abstract

**Highlights:**

**What are the main findings?**

- Existing ordinary differential equation models incorporate no more than two regulated cell death types simultaneously, limiting their capacity to capture the full complexity of pathway crosstalk and coordination.
- Ferroptosis is currently modeled only as an independent process, with no mechanistic links established to other regulated cell death pathways.
- Comprehensive models that integrate molecular mechanisms of regulated cell death, damage-associated molecular pattern release, and subsequent intercellular immune activation are still absent.

**Abstract:**

Mathematical modeling is essential for understanding the complex regulatory pathways governing cell death and survival, including apoptosis, necroptosis, pyroptosis, ferroptosis, autophagy, and immunogenic cell death (ICD)—a functional category comprising diverse morphological types capable of activating immune responses. The growing number of models describing individual signaling pathways poses the challenge of integrating them into a cohesive framework. This review aims to identify common components across existing ordinary differential equation models that could serve as key nodes to merge distinct signaling modalities. Proposed models highlight Bcl-2, Bax, Ca^2^, and p53 as shared regulators linking autophagy and apoptosis. Necroptosis and apoptosis are interconnected via TNF signaling network and modulated by caspase-8, c-FLIP, and NFκB, with RIPK1 acting as a critical hub directing pathway choice. Pyroptosis and apoptosis are co-regulated by NFκB, tBid, and caspases, while ferroptosis is modeled exclusively as an independent process, separate from other forms of cell death. Furthermore, existing models indicate that ICD intersects with necroptosis during oncolytic virotherapy, with pyroptosis in SARS-CoV-2 infection, and with apoptosis in the context of chemotherapy. Although several models address crosstalk between pairs of cell fate decisions, creating comprehensive frameworks that encompass three or more death modes remains an open challenge.

## 1. Introduction

The fundamental principle of organismal function is homeostasis, defined as the ability to maintain a stable internal functional and structural state despite external changes [1]. At the cellular level, homeostasis is achieved through a delicate balance between cell proliferation (division and growth) and regulated cell death (RCD) [2,3]. Advances in elucidating the molecular mechanisms of cell death have led to the establishment of the Nomenclature Committee on Cell Death (NCCD), which regularly updates recommendations for defining and interpreting cell death from morphological, biochemical, and functional perspectives [4,5,6,7,8]. The current RCD types include necroptosis, ferroptosis, alkaliptosis, apoptosis, autophagy-dependent cell death, cuproptosis, disulfidptosis, entosis, immunogenic cell death (ICD), lysosome-dependent cell death, mitochondrial permeability transition (MPT)-driven necrosis, necroptosis, NETosis, oxeiptosis, parthanatos, and pyroptosis. Each modality is driven by specific molecular pathways characterized by extensive interconnectivity [7,9,10]. Alongside molecular discoveries, mathematical modeling has become indispensable for quantifying, predicting, and analyzing cell dynamics across diverse conditions [11]. Ordinary differential equations (ODEs) are a widely used approach for representing biological processes [12,13]. However, modeling cellular behavior extends well beyond ODEs [14] to include approaches such as partial differential equations [15,16,17,18,19], Boolean networks [20,21,22,23,24,25,26], cellular automata [27,28], and agent-based simulations [29,30]. Comprehensive reviews have been published on models of apoptosis [31,32,33], autophagy [34,35], and ICD [36,37,38], along with a notable review that integrates models across these and other cell death pathways [39]. Despite this progress, the increasing number of models describing individual signaling modalities poses the challenge of integrating them into a single framework capable of capturing the complexity of cell death regulation. This challenge resonates with Michael Savageau’s concept that every reductionist must also act as a reconstructionist, implying that a true understanding of complex biological systems involves not only breaking them down into fundamental parts but also reassembling those parts to uncover the emergent behaviors [40].

Systematic efforts have been made to identify shared components across various cell death and survival pathways, aiming to develop a unified mathematical framework for cell fate decisions [23,29,41,42,43,44,45,46,47]. However, existing ODE-based models consider no more than two RCD types simultaneously. This overview addresses this limitation by initiating steps toward the integration of multiple RCD pathways within a unified ODE model. Among the regulatory pathways controlling cell death and survival, ODE models have been developed for apoptosis, autophagy, ferroptosis, ICD (primarily involving intercellular interactions), necroptosis, and pyroptosis. Since all existing crosstalk models incorporate apoptosis with other death modalities, we begin our exploration with apoptosis models and then examine other pathways, highlighting hubs of their intersection with apoptosis and, where data allow, with additional RCD types. Characteristics of all models discussed in this review are summarized in Supplementary Table S1 and can be accessed online via the GitLab wiki at https://gitlab.sirius-web.org/virtual-cell/info/-/wikis/Cell%20fate/Home (accessed on 11 November 2025).

## 2. Apoptosis

Apoptosis is a universal, evolutionarily conserved form of RCD involving caspase family proteases [8]. It plays key roles in eliminating unwanted cells during development, removing damaged or infected cells, and maintaining tissue homeostasis by controlling cell populations [48]. The process of apoptosis comprises three distinct phases: induction (signaling), effector, and degradation [49,50]. The induction phase is triggered by external signals, such as death receptor (DR) activation, or internal stimuli like mitochondrial stress, initiating the caspase cascade [51,52]. Caspases are synthesized as inactive zymogens, known as procaspases, and are classified into two types depending on their roles in apoptosis: initiator (caspases-2, -8, -9, and -10) and effector (caspases-3, -6, and -7) [53,54]. During the effector phase, the proteolytic cleavage of vital cellular proteins orchestrates apoptotic dismantling [55]. The degradation phase culminates in cellular fragmentation into membrane-bound apoptotic bodies that are quickly phagocytosed by neighboring cells, including macrophages and parenchymal cells [56].

Death receptors (DRs) belong to the tumor necrosis factor (TNF) receptor superfamily, including TNFR1 (p55/CD120a), TNFR2 (p75/CD120b), CD95 (Fas/APO-1), and TNF-related apoptosis-inducing ligand (TRAIL)-R1 (DR4) and -R2 (DR5/APO-2) [57]. The stimulation of CD95 and TRAIL-R1/R2 with their cognate ligands leads to the assembly of the death-inducing signaling complex (DISC), which, in addition to the receptor, includes the Fas-associated protein with death domain (FADD), procaspases-8/-10, and cellular FADD-like interleukin (IL)-1β-converting enzyme-inhibitory proteins (c-FLIPs). The DISC serves as a platform for procaspase-8 processing and activation [58]. In contrast, the TNFR1-induced pro-apoptotic pathway requires the formation of two distinct complexes. The membrane-bound complex I, which is formed rapidly, consists of TNFR1, TNFR1-associated death domain protein (TRADD), receptor-interacting protein kinase 1 (RIPK1), TNF receptor-associated factor 2 (TRAF2), cellular inhibitor of apoptosis proteins (cIAPs), LUBAC complex [59], and inhibitory κB (IκB) kinases (IKKs) and triggers nuclear factor κB (NFκB) response, but not apoptosis. This complex gives rise to a cytosolic complex II, which lacks TNFR1 but includes RIPK1, FADD and procaspases-8/-10 to initiate apoptosis. However, cell death is generally not a typical outcome, as NFκB induces the transcriptional regulation of c-FLIP, the main inhibitor of caspase-8 as well as other inhibitors of apoptotic pathway [60,61,62].

The intrinsic apoptotic pathway is triggered by stress stimuli or extrinsically via caspase-8-induced proteolysis of Bid into tBid and activation of Bax [63], leading to mitochondrial release of cytochrome c and Smac, which activate caspase-9 [64]. Subsequent to this, active caspases-8 and -9 cleave caspases-3 and -7 [65], thereby initiating the cleavage of poly (ADP-ribose) polymerase-1 (PARP-1) [66,67]. The primary function of PARP-1 is to detect and repair DNA damage. Consequently, following its cleavage, the process of apoptosis becomes irreversible [67,68]. This pathway is blocked by anti-apoptotic Bcl-2 family members (Bcl-2, Bcl-xL), which prevent mitochondrial release of apoptogenic factors and regulate Bax activation [69,70,71]. Depending on the pathway of apoptosis caused by DRs, cells are classified as type I, where caspase-8 directly activates effector caspases, or type II, where this activation requires mitochondrial involvement [72,73,74,75,76].

In 2000, Fussenegger et al. pioneered a model capturing key aspects of receptor-mediated and stress-induced caspase activation [77]. Since then, apoptosis modeling has become widespread within the scientific community. While several research groups following Fussenegger and colleagues have simulated the interplay between intra- and extracellular signals that initiate apoptosis [78,79,80,81], other studies (reviewed below) have focused more narrowly on pathways related to specific triggers, such as DRs or intracellular catastrophes.

Bentele et al. developed a detailed computational model of CD95 signaling, revealing a threshold mechanism whereby c-FLIP inhibits caspase-8 activation at the DISC under low ligand conditions, blocking apoptosis [82]. The model was calibrated using experimental data from SKW 6.4 human B lymphoblastoid cells, which were classified as type I due to elevated DISC formation [74]. Hua and colleagues next constructed a model that reproduced the transition from type II (Bcl-2–sensitive) to type I (Bcl-2–insensitive) signaling by increasing caspase-8 levels [83]. Simplified versions of this model were used in later studies [84,85]. Neumann et al. proposed an integrated quantitative model of CD95-mediated apoptosis and NFκB activation, uncovering a novel mechanism in which p43-FLIP (a c-FLIP cleavage product generated by procaspase-8 at the DISC) directly interacts with the IKK complex leading to its activation, a finding validated experimentally [86]. In the same year, Fricker et al. furthered understanding of c-FLIP isoform roles (c-FLIP long, c-FLIP_L_; c-FLIP short, c-FLIP_S_; and c-FLIP Raji, c-FLIP_R_) in caspase-8 activation at the DISC by constructing a mathematical model of this process [87]. The findings demonstrated that c-FLIP_L_ promotes apoptosis only at moderate expression in combination with strong receptor stimulation or in the presence of high amounts of one of the short c-FLIP isoforms, c-FLIP_S_ or c-FLIP_R_. In subsequent research, Kutumova and colleagues reduced and combined the Bentele and Neumann models into a comprehensive computational framework for the CD95 and NFκB pathways, suitable for analyzing pro- and anti-apoptotic signaling in both type I and type II cells [88]. This framework was recently integrated with a mechanistic cardiorenal model [89,90,91], enabling in silico exploration of age-dependent cardiomyocyte apoptosis and its systemic effects [92]. Models of CD95-induced apoptosis developed in the last decade have provided new insights into the molecular mechanisms of the CD95 signaling network. Wu and Finley developed a model of Thrombospondin-1 (TSP1)-mediated apoptosis [93] by adapting molecular details of the CD95L cascade from the model by Neumann et al. [86]. The Lavrik group investigated cell-to-cell variability in the apoptotic phenotype and showed that it arises not from stochastic gene expression in the NFκB response, but from heterogeneity in the composition of death effector domain (DED) chains (filaments) formed by procaspase-8 within the DISC [94]. Subsequent models created by the group further clarified the mechanisms underlying caspase-8 activation at the DED filaments as well as their regulation [95,96]. Finally, Mangrum and Finley extended the caspases-mediated apoptosis model originally established by Eissing et al. [97] by incorporating extracellular activation and CD95 receptor dynamics, identifying key modulators of apoptosis in heterogeneous cancer cell populations that may be leveraged to enhance cell death [98].

Several models have been developed to study TRAIL signaling [99,100,101,102,103]. Albeck et al. showed that the delay before effector caspase activation depends on caspase-8 activity and the rates of downstream reactions immediately following TRAIL stimulation [99,100]. The findings of this study indicate that interactions between Bcl-2 family proteins, the segregation of Smac from XIAP (X-linked IAP), and mitochondrial pore formation mechanisms are critical for the snap-action switch that enforces a binary decision between cell survival and death. Zhang and coworkers adapted Hua’s model [83] to TRAIL signaling and revealed that high expression of decoy receptors (TRAIL-R3 and TRAIL-R4) relative to DRs (TRAIL-R1 and TRAIL-R2) negatively regulates apoptosis via ligand-independent binding [101]. The intricate mechanism through which decoy receptors can disrupt the assembly of TRAIL receptor homotrimers was modeled by Laussmann and colleagues [102]. The authors demonstrated that caspase-8 activation might be delayed in the presence of proteasome inhibitors, particularly at submaximal doses of TRAIL. Anderson et al. developed a simplified model of TRAIL-induced apoptosis that included only the key reactions to investigate the systemic effects of protein kinase B (AKT) [103]. The model predictions clarified how individual protein species, as well as the apoptotic system in its entirety, are influenced across different genetic backgrounds. Subsequent laboratory studies revealed that AKT regulation of apoptosis is significantly stronger during TRAIL-mediated extrinsic apoptosis compared to TRAIL-independent pathways, with its effects most pronounced in the early stages of the apoptotic response.

The pioneering model of the cellular decision between apoptosis and proliferation in response to TNFα stimulation was established by Cho et al. [104]. Despite its simplicity and lack of quantitative calibration with experimental data, their systems-theoretical approach captured the ambiguous outcomes of TNFα signaling. Rangamani and Sirovich expanded this model by incorporating NFκB-driven transcription of IAP and IκB, activation of caspases-8 and -3, and DNA fragmentation [105]. Koh and Lee proposed an even more complex model that included the intrinsic apoptotic pathway alongside the extrinsic one, as well as NFκB-mediated synthesis of apoptosis regulators A20 and c-FLIP [106]. The critical roles of A20 and c-FLIP in modulating TNFα responses were also explored in an experimentally validated mathematical model of TNFR1-induced pro- and anti-apoptotic pathways by Schliemann et al. [107]. Most recently, Halder and Chatterjee designed a novel model describing the bistable switch between cell survival and death triggered by TNFα, elucidating the mechanism of TNFR2 signaling in T-regulatory cells that suppress immunological responses to preserve homeostasis and self-tolerance [108].

Computational models with a focus on mitochondria-dependent apoptotic pathways have been developed by multiple research groups, taking into consideration a variety of stimuli that initiate the process. Bagci et al. simulated the effects of silymarin [109], which elevates p53 levels, downregulates Bcl-2, and upregulates Bax [110]. This model was later expanded to include nitric oxide–induced reactions, revealing its complex, competing roles in apoptosis regulation [111]. The calculations indicated that the balance between pro- and anti-apoptotic outcomes depends on the relative concentrations of reactive nitric oxide species and their interactions with glutathione, with precise timing of nitric oxide production and external stimulation being crucial. Another extension of the Bagci model, involving the p53-Mdm2 feedback loop and p53-induced G2/M phase cell cycle arrest, demonstrated that intrinsic apoptosis triggered by severe DNA damage is driven by oscillatory p53 dynamics and initiated strictly after cell cycle arrest [112]. Zhang et al. clarified this scheme by proposing that cell cycle arrest coordinates DNA repair, and apoptosis ensues if the damage is irreparable [113]. Their model attributed this cell-fate transition to a transformation of p53 from a “helper” (pro-arrest) to a “killer” (pro-apoptotic) form. Notably, the timing of p53 activation is critical, as DNA-damaging agents can both induce p53-driven apoptosis and promote apoptosis inhibitors that counteract cell death [114].

To study the mechanisms behind fractional killing by DNA-damaging chemotherapeutic agents, Ballweg et al. constructed a mathematical model representing this complex biological process [115]. Their analysis predicted that cell fate depends on the interplay between the temporal trajectories of p53 and cIAP and the bifurcation geometry that defines how Bax is activated by these two control parameters. McKenna and colleagues further explored how DNA-damaging drugs trigger apoptosis and highlighted Bax and Smac as key regulators of the apoptotic switch, with Bax acting as a primary mediator of mitochondrial outer membrane permeabilization (MOMP) [116]. Their findings complement the research of Rehm et al. [117], who identified MOMP as the essential initiating event in intrinsic apoptosis and developed a biochemical model based on experimental data obtained in HeLa cells that accurately reproduced the rapid in vivo kinetics of effector caspase activation following staurosporine treatment. Moreover, the crucial role of Smac concentrations was explored for the effective apoptosis initiation. In contrast, Ryu et al. focused specifically on apoptosome formation as a critical step in apoptosis [118], parameterizing their model using experimental data from Rodriguez and Lazebnik [119], where activation of caspases-9 and -3 was induced in HEK293 cell extracts by ATP or dATP. Finally, other models abstract from specific stimuli that initiate mitochondrial apoptosis and instead concentrate on the ensuing molecular interactions, thereby providing deeper insights into the dynamic regulation and bistable properties of the process [27,120,121,122].

In summary, although existing models capture specific aspects of the apoptotic signaling network, a comprehensive model that integrates all apoptosis scenarios remains unavailable. Despite the efforts to develop such a model [123], the primary challenge lies in the lack of sufficient experimental data for its calibration.

## 3. Autophagy

Macroautophagy (commonly referred to as autophagy) is a process where cellular contents are encircled by the membrane-bound structures called autophagic vesicles. These vesicles subsequently fuse with lysosomes, where the cellular contents are broken down into small molecules, which are recycled by the cell [124,125]. The modeling of autophagy has a long history. As early as 1975, Russell Deter proposed a conceptual model describing the interactions among telolysosomes, autophagosomes, and autolysosomes during glucagon-induced autophagy in rat liver [126]. More recently, Han and colleagues applied similar approaches to mammalian hepatocytes [127] and human neurons [128], modeling autophagosome formation from proteins and organelles and subsequent autolysosome formation. These studies identified intracellular ATP and amino acid levels as key biochemical parameters reflecting cellular energetic and metabolic states that influence cell fate. Using combined computational and experimental methods, Dalle Pezze et al. investigated how amino acids regulate core autophagy controllers: AMP-activated protein kinase (AMPK) and Unc-51-like kinase 1 (ULK1), which promote autophagy, and the mammalian target of rapamycin (mTOR) complex 1 (mTORC1), which inhibits it [129]. They found that amino acids acutely activate AMPK concurrently with mTOR, and in the presence of sufficient amino acids, AMPK does not inhibit mTORC1 but sustains ULK1 activity and autophagy.

Autophagy dysfunction is associated with a broad spectrum of pathologies, including cancer, neurodegenerative conditions, metabolic disorders, and autoimmune diseases [130,131,132,133]. Due to their pivotal functions, the kinase triad AMPK, MTORC1 and ULK1 represents a promising target for pharmaceutical interventions [134,135,136]. Reflecting this, several mathematical models have recently been developed to simulate the effects of drugs targeting these key autophagy regulators [137,138,139,140,141].

In 2011, Tyson et al. designed a roadmap for a pioneering ODE model of estrogen receptor signaling in breast epithelial cells that, for the first time, integrated the interplay between autophagy and apoptosis in response to stress activating the unfolded protein response (UPD) [41]. They emphasised that under low stress conditions, autophagy promotes cell survival; at moderate stress, it may lead to autophagic cell death; and for large stress, calcium release may stimulate apoptosis by the intrinsic (mitochondrial) pathway. Subsequently, the Tyson group created a dynamic model that was consistent with quantitative measurements of autophagy and apoptosis in rat kidney proximal tubular cells in response to cisplatin-induced stress [142]. Concurrently, Cook et al. presented a mathematical model which, together with experimental data, demonstrated that knockdown of estrogen receptor-α in antiestrogen-resistant cells stimulates mitochondrial dysfunction and inhibits the UPR-mediated antioxidant pathway, thereby promoting reactive oxygen species formation and apoptosis [143].

Autophagy and apoptosis share common regulators, with Bcl-2 playing a central role in their crosstalk [144,145]. To explore the physiological relevance of this interaction, Kapuy et al. developed a computational model incorporating Bcl-2, Bax, the autophagy inducer Beclin-1, and caspases [146]. Subsequent studies from the Kapuy group examined survival and death control through a series of minimalistic models. Specifically, the initial model of the regulatory triangle involving AMPK, mTORC1, and ULK1 [147] was based on computational analysis of a bistable switch between autophagy and protein synthesis at the translational level, originally described by Szymańska and colleagues [148]. This was followed by multiple models investigating AMPK–mTORC1–ULK1 network behavior [149,150,151,152,153,154]. Additional regulators were also considered, including the transcription factor NRF2 (nuclear factor erythroid-related factor 2) [150,155], sensors for endoplasmic reticulum (ER) stress such as PERK (protein kinase RNA-like ER kinase) and IRE1 (inositol requiring 1) [156], PERK-induced protein ATF4 (activating transcription factor 4) and its two targets, GADD34 (growth arrest and DNA damage-inducible 34) and CHOP (C/EBP homologous protein) [150,157,158]. The effects of diverse chemicals on cell death mechanisms were also examined, including sulforaphane [153], tropic antimalarial drugs (chloroquine, hydroxochloroquine) after SARS-CoV-2 infection [159], and chloroquine combined with pharmacologic ascorbate in KRAS mutant tumors [160]. It was demonstrated that ER stress generates an autophagy-dependent apoptotic threshold, and that double-negative feedback between autophagy and apoptosis inducers can yield bistability [161,162]. This extensive body of work was complemented by studies from Yang and co-authors. Using the primary Kapuy model [146], Yang et al. investigated the binding of the cancer cell regulator AMBRA1 (Beclin1-regulated autophagy protein 1) [163] to Bcl-2 in the ER and mitochondria under varying stress levels [164]. They showed that AMBRA1 competes with Bcl-2 family proteins to activate Beclin-1, thereby promoting sustained autophagy prior to apoptosis. Furthermore, noise effects on the double-negative feedback between autophagy and apoptosis were examined using a minimal three-component model of stress, caspases, and Beclin-1, revealing that stronger noise increases randomness in switching [165]. Finally, extending the model of cell fate decisions during ER stress [161], Yang et al. explored autophagic degradation of misfolded α-synuclein (whose accumulation is associated with Parkinson’s disease) and found that oxidative stress can transform the bistable switch of misfolded α-synuclein aggregation from an irreversible to a reversible condition through the autophagy-lysosome degradation pathway [166].

The interplay between autophagy and apoptosis relies heavily on calcium-dependent signaling pathways [167,168]. The role of Ca^2+^ as a rheostat fine-tuning cellular responses was investigated in a comprehensive model by Liu et al. [44], which incorporates mTOR and inositol signaling, as well as the autophagic and intrinsic apoptosis pathways. Their study highlighted that, beyond Ca^2+^ released from the ER, p53 also acts as a crucial determinant of cell fate through its regulation of Bcl-2, Bax, AMPK, and damage-regulated autophagy modulator (DRAM), a lysosomal protein that induces autophagy. Since the authors did not release the source code for their model and could not provide it upon request, Hajdú et al. reconstructed, calibrated, and made the model publicly available [169]. Additionally, several research groups have examined the complex roles of calcium ions and p53 in autophagy-apoptosis decisions using simpler models [41,43,142,170,171].

Finally, in addition to the extensively studied regulators of autophagy and apoptosis described above, mathematical modeling has been applied to explore more specific signaling pathways influencing cell fate decisions. For instance, Schwartz-Roberts et al. reported an inverse correlation between the expression of nuclear interferon regulatory factor-1 (IRF1) and the autophagy regulator ATG7 in human breast cancer cells [172]. Using a computational model to generate signaling hypotheses, they provided evidence that ATG7 silencing could resensitize IRF1-attenuated cells to apoptosis via mechanisms involving other estrogen-regulated genes. Furthermore, Pavel et al. constructed a model of the mutual regulation between the Hippo pathway and autophagy [173].

## 4. Ferroptosis

Ferroptosis is a recently discovered form of cell death characterized by the lethal accumulation of iron-dependent membrane-localized lipid peroxides [174,175,176,177]. Although apoptosis and ferroptosis mechanisms were initially thought to be mutually exclusive, current studies have revealed cellular contexts requiring a balanced interaction between these death pathways [178,179,180]. Despite intensive research conducted in this field in recent times, computational models capable of adequately characterizing the switch between ferroptosis and apoptosis remain elusive. Moreover, a review of the extant literature indicates that only three ODE models of ferroptosis have been developed to date [181,182,183].

The first model of the phospholipid metabolic network regulating ferroptosis was constructed by Kagan et al. in 2017 [181]. The model replicated glutathione peroxidase 4 (GPX4) deficiency induced by its inhibitor RSL3 and triggering ferroptosis and contributed to the discovery that ferroptosis execution involves a highly organized oxygenation center, where only one class of phospholipids (phosphatidylethanolamines) undergoes oxidation in ER-associated compartments with the specificity towards two fatty acyls: arachidonoyl and adrenoyl. Furthermore, vitamin E was shown to regulate ferroptosis by suppressing lipoxygenases involved in death signaling, thereby suggesting its protective role [181].

The next model created by the Kagan group was designed to enhance comprehension of the nitric oxide (NO•) effects on the ferroptotic program of macrophages/microglia [182]. In computational experiments, elevated expression of inducible nitric oxide synthase (iNOS) decreased sensitivity of macrophages to RSL3 stimulation. Concurrently, both inhibitory mechanisms of NO•, including the inactivation of 15-lipoxygenase and the scavenging of lipid radical intermediates, was significant for iNOS-mediated suppression of RSL3-stimulated ferroptosis in activated M1 cells.

The most recent model by Arbatskiy et al. presents a comprehensive modular framework for ferroptosis, integrating the key molecular and genetic mechanisms that regulate its execution: Fenton’s reaction, iron metabolism, lipid synthesis and peroxidation, the pentose phosphate pathway, and the antioxidant system, which encompasses both the gamma–glutamyl cycle and the GPX4 system [183].

## 5. Immunogenic Cell Death

ICD is a form of RCD that activates an adaptive immune response against dying cells, in particular cancer cells [184,185,186,187]. ICD can be initiated by a wide range of stimuli, including viral infections, chemotherapeutic agents, physicochemical therapies, photodynamic therapy, and radiotherapy [7,188]. The primary forms of ICD are pyroptosis, necroptosis, and ferroptosis, which promote immune activation by releasing damage-associated molecular patterns (DAMPs), while apoptosis is typically regarded as an immunologically silent process [3,189]. However, under certain conditions (such as exposure to chemotherapy or radiation), apoptosis can acquire immunogenic properties [190,191,192].

Mathematical modeling of ICD has been refined over the years in the context of anticancer therapies, aiming to enhance understanding and optimize immune-mediated treatment outcomes. As early as 1977, DeLisi and Rescigno introduced a predator-prey model of carcinogenesis that described the dynamics of tumor cells in the presence of tumor-stimulated killer lymphocytes [193,194]. In the proposed model, the survival probability of a solid tumor increased with tumor size and depended on both the timing of immune intervention and the status of the recipient’s immune system. It was much more sensitive to variations in tumor characteristics, such as antigenicity, than to alterations in lymphocyte parameters. However, the “sneaking through” phenomenon, defined by the progressive growth of small antigenic tumors, rejection of medium-sized tumors, and eventual breakthrough of large tumors [195,196], was not observed. The model developed by Grossman and Berke [197] specifically examined this phenomenon and was subsequently complemented by a series of in silico studies that delved deeper into the mechanisms underlying tumor rejection and breakthrough [198,199,200]. In 1994, Kuznetsov et al. introduced a mathematical model depicting the cytotoxic T lymphocyte response to the progression of immunogenic tumors [201], which has become a widely accepted standard in the field of mathematical oncology [36,37,202]. This model captures key in vivo phenomena, such as tumor dormancy, immunostimulation of tumor growth, and tumor sneaking through, analyzing steady states, bifurcations, and oscillatory behaviors in tumor-immune dynamics.

Although models of initial antitumor immune activation continued to emerge [203,204], since the late 1990s, ICD modeling has advanced to incorporate more sophisticated mechanisms of tumor regulation, including the influence of the tumor microenvironment [205,206,207,208], immune-related factors driving tumor regrowth [209], metastatic lesion [210], and responses to therapy. The evolution of models for studying ICD inducers had closely reflected the development of therapeutic strategies. To date, these models encompass major immunotherapy modalities, including immune checkpoint inhibitors [211,212,213,214,215,216,217,218,219,220,221], cancer vaccines [222,223,224,225], monoclonal antibodies [223], adoptive cell transfer [219,222,226,227], immune system modulators [219,222,226,228,229,230,231], and oncolytic viruses [215,220,224,232,233]. A number of models have also investigated the complex effects of other cancer therapies on the immune system, including radiotherapy [210,211,212,214,217,218,234,235,236,237,238,239], chemotherapy [221,222,227,240,241], and surgical interventions [223,235,236]. The prevailing consensus from these models is that although monotherapies can lead to promising outcomes in some cases (especially in early-stage cancer), combination therapies, involving multiple immunotherapeutic agents or integrating immunotherapy with chemotherapy, radiotherapy, surgery, or targeted therapy are generally more effective [211,212,214,215,216,217,218,219,220,222,223,224,226,227]. This is particularly relevant given that approximately 50% of cancers are diagnosed at advanced stages [242]. Moreover, integrating computational models of the tumor microenvironment with clinical patient data can facilitate the development of personalized combination therapies, leading to more effective treatment regimens than standardized protocols [221]. This tailored approach has the potential to improve disease control, increase survival rates, and minimize side effects, ultimately optimizing overall patient outcomes.

In conclusion, it is important to highlight that among the models discussed, only one explicitly attributes that immune activation occurs in response to apoptosis of bladder cancer cells induced by mitomycin-C chemotherapy, while avoiding molecular complexities [241]. The other models do not specify a particular cell death mechanism responsible for ICD.

## 6. Necroptosis

Necroptosis is a form of RCD that critically depends on RIPK1, RIPK3 and the mixed lineage kinase domain-like (MLKL) pseudokinase and is typically characterized by morphological features resembling necrosis [243,244,245]. This pathway can be initiated by the activation of DRs, with TNFR1 being the most extensively studied, particularly in the contexts of caspase-8 or FADD deficiency, or in the presence of the caspase inhibitor Z-VAD-FMK [246,247,248]. In contrast, the catalytic activity of the caspase-8–c-FLIP_L_ complex acts to inhibit necroptosis [249].

The computational modeling of necroptosis is currently in its nascent stage. In 2020, Oliver Metzig and colleagues introduced a conceptual mathematical model to investigate the complex interplay between TNF-induced NFκB signaling and necroptotic fate decisions [250]. Their research identified the TNF-induced, NFκB-responsive protein A20 as a critical regulator whose expression forms an incoherent feedforward loop, protecting a fraction of cells from transient TNF doses but sensitizing them to prolonged TNF stimulation. Moreover, they found that dysregulated NFκB dynamics diminish the cellular discrimination of TNF exposures. Xu et al. explored oscillations within the necroptotic network by analyzing a circuit of four key components: TRADD, RIPK1, caspase-8, and RIPK3 [251]. They demonstrated that elevated levels of RIPK1 and caspase-8 increase the probability of oscillations, while high TRADD levels reduce it. Their study revealed that the minimal essential structure for generating oscillations is a paradoxical component combined with positive feedback among RIPK1, caspase-8, and RIPK3. Ildefonso et al. further examined the biochemical regulation of necroptosis, focusing on a critical activation step–RIPK1 deubiquitination mediated by the enzymes CYLD (cylindromatosis lysine 63 deubiquitinase) and A20, whose precise roles have been debated [252]. To clarify these contradictions, they constructed a detailed model of TNF-induced necroptosis and identified four distinct signaling modes. In one mode, A20 and CYLD work together to drive RIPK1 deubiquitination; in another, CYLD acts as the sole deubiquitinase; while in the remaining two modes, either enzyme serves as the driver, with the other unexpectedly inhibiting necroptosis. This model reconciles conflicting and counterintuitive experimental findings across various cell types by associating them with these necroptosis modes.

Beyond models specifically focused on necroptosis, several have been developed to explore the interplay between apoptosis and necroptosis. Li et al. addressed the paradoxical role of RIPK1, which can both promote and limit necroptosis [45]. Combining mathematical modeling with experimental validation, they found two critical RIPK1 molecule thresholds per cell: a lower threshold of approximately 1000 molecules and an upper threshold of approximately 46,000 molecules. Below the lower threshold, cells undergo only TRADD-dependent apoptosis. Between the thresholds, the recruitment of procaspase-8 and RIPK3 to the necrosome (dependent linearly and nonlinearly on RIPK1 levels, respectively) explains the coexistence of apoptosis and necroptosis as well as RIPK1’s contradictory functions. Above the upper threshold, apoptosis is suppressed, resulting in necroptosis alone. These findings highlight the complex interplay of proteins in TNF signaling, demonstrating how RIPK1 orchestrates diverse cell fate decisions. More recently, Lee et al. developed a computational model depicting the interactions between tumor cells and therapeutic agents such as bortezomib and oncolytic viruses, which induce both necroptosis and apoptosis by modulating an intracellular signaling network comprising NFκB, IκB, Bax, and RIPK1 [253]. The model effectively integrates the dynamics of cell–cell and cell–drug interactions (an approach typical of ICD modeling) with intracellular processes, thereby providing valuable insights to optimize clinical protocols in cancer therapy.

Taken together, these models clarify the regulation of necroptosis and its close interconnection with other cell death pathways, demonstrating that RIPK1 not only serves as a distributor directing signals toward apoptosis or necroptosis but also actively influences the selection of the specific pathway.

## 7. Pyroptosis

Pyroptosis is an inflammatory form of RCD characterized by cell membrane rupture and the release of interleukin (IL)-1β and IL-18 [254,255,256,257,258,259,260,261]. This process is primarily mediated by inflammatory caspases-1, -4, -5, and -11 that cleave gasdermin D (GSDMD). The resulting N-terminal fragment of GSDMD forms pores in the cell membrane, causing cell swelling and lysis. Additionally, caspase-3 can cleave gasdermin E (GSDME), shifting apoptosis toward pyroptosis via a similar pore-forming mechanism that culminates in membrane rupture [262,263].

Although the term “pyroptosis” was first introduced in 2001 [264,265], mathematical modeling of pyroptotic signaling pathways has only recently begun to emerge. To date, just a few ODE models explicitly characterize this phenomenon [46,47,266,267]. Among these, the model developed by Hamis and Macfarlane [266] simulates pyroptosis within a single cell following SARS-CoV-2 infection, detected by toll-like receptors (TLRs) on immune and lung epithelial cells [268]. Upon recognition of viral pathogen-associated molecular patterns (PAMPs), TLRs induce the translocation of NFκB to the nucleus, triggering synthesis of inactive NLRP3 (NLR family pyrin domain containing 3) and pro-IL-1β (the IL-1β precursor). Various stimuli, such as ionic flux or excessive reactive oxygen species, activate NLRP3, causing it to oligomerize and form the inflammasome base, which recruits ASC (apoptosis-associated speck-like protein containing a CARD) and procaspase-1. Within this complex, procaspase-1 becomes activated and cleaves GSDMD, pro-IL-1β, and pro-IL-18 into their active forms, initiating pyroptotic cell death. The model also incorporates anti-inflammatory therapy that inhibits NLRP3 inflammasome formation, demonstrating how such intervention can redirect cell death from inflammatory pyroptosis to non-inflammatory pathways like apoptosis, thereby preventing uncontrolled cytokine release.

IL-1β and IL-18 are potent proinflammatory cytokines [269,270,271,272,273] whose release during pyroptosis underscores its role as a highly immunogenic form of cell death. From this perspective, the Hamis-Macfarlane model can be integrated with existing mathematical models of ICD described above. Although these ICD models were primarily designed to study tumor-immune interactions, they are adaptable to virus-infected cells due to shared biological mechanisms underpinning anticancer and antiviral immunity. Moreover, some models developed for oncolytic virus therapy already incorporate viral dynamics alongside immune responses, demonstrating their flexibility to model viral ICD [215,220,224,232,233]. Integrating the intracellular molecular mechanisms of pyroptosis with the intercellular processes considered by ICD models into a unified framework offers a promising strategy to deepen our understanding of the complex mechanisms driving severe SARS-CoV-2 manifestations and to inform the development of preventive interventions.

To investigate the interplay between pyroptosis and apoptosis, Yin and colleagues constructed a coarse-grained model of Salmonella Typhimurium-induced caspase-1 activation, which drives two competing pathways: GSDMD cleavage leading to pyroptosis, and activation of caspases-8, -9, and -3 triggering apoptosis [46]. Using sensitivity and bifurcation analyses, they explored how the network switches between cell death modes, identifying conditions that produce monostability, bistability, and tristability. Their findings revealed that only changes in caspase-1 or GSDMD levels can independently switch cell fate, with reductions in either favoring a shift from pyroptosis to apoptosis. A similar approach was later applied to create a model of pyroptosis and secondary pyroptosis by expanding the refined network of caspases-1, -3, -8, -9, and GSDMD to include two additional components: tBid, which mediates caspase-1-induced activation of caspase-9, and GSDME, cleaved by caspase-3 to initiate secondary pyroptosis [267]. Additionally, Li et al. proposed a model of NLRP1b inflammasome signaling that integrates the downstream cascade involving ASC, caspases-1, -3, -8, -9, and GSDMD, capturing possible pyroptosis and apoptosis outcomes [47]. Through combined modeling and experimental analysis, they showed that pyroptosis switches to apoptosis at a very low caspase-1 threshold but requires a high GSDMD threshold. Caspase-1-impaired cells activate apoptosis via an ASC-caspase-8-dependent pathway, while GSDMD-deficient cells primarily rely on a caspase-1-dependent route. Moreover, caspase-1 and GSDMD can independently trigger the simultaneous occurrence of pyroptosis and apoptosis. Together, these models reveal key regulatory mechanisms governing cell death decisions and highlight potential targets for therapeutic intervention.

## 8. Comparative Statistics

We aimed to provide a comprehensive overview of ODE models describing various cell death modalities. Our focus was on ODE modeling as the predominant mathematical approach for capturing complex signaling networks. The summary covered 137 ODE-derived mathematical models of cell death and survival (Supplementary Table S1), comprising 40 apoptosis models, 20 autophagy models, 46 ICD models, 3 ferroptosis models, 3 necroptosis models, 2 pyroptosis models, and 23 hybrid models involving multiple pathways. Among the hybrid models, 19 combine apoptosis and autophagy, 2 integrate apoptosis and necroptosis, and 2 link apoptosis with pyroptosis. Program code is publicly available for 69 models, accounting for 50% of the total (Table 1). The most used modeling formats are MATLAB (45 models), SBML (27 models), and XPPAUT (22 models). Additionally, 23 of the 27 SBML models are accessible through the BioModels database [274,275].

## 9. Integrated Model of the Pathways

In summary, our analysis of the developed models revealed key common elements that integrate diverse cell death pathways into a unified decision-making framework governing cell fate. It is also worth to note that the framework does not consider the ferroptosis module due to the state-of-the-art knowledge and data in the field justifying the ferroptosis does not have any mechanistic relationships established to other regulated cell death pathways.

At the core of the crosstalk between apoptosis and autophagy lies the Bcl-2 family of proteins, notably Bcl-2 and Bax. Bcl-2 serves as a crucial regulator by inhibiting Bax-mediated apoptosis, primarily by preventing the mitochondrial release of apoptogenic factors such as cytochrome c and Smac [83,85,99,100,101,103,109,112,113,120]. Additionally, Bcl-2 modulates autophagy through its interaction with the autophagy initiator Beclin-1 [41,43,44,142,146,164,170,171]. Calcium signaling acts as a rheostat in this balance; calcium ions released from the endoplasmic reticulum influence both autophagic and intrinsic apoptotic pathways via modulators like BH3, mTOR and inositol signaling components [43,44,142,170]. The tumor suppressor p53 has a dual regulatory role, promoting apoptosis by controlling Bcl-2 and Bax, while also modulating autophagy through AMPK inhibition and DRAM activation [44,171].

Both apoptosis and necroptosis are triggered by activation of DRs from the TNF receptor superfamily, which assemble multi-protein signaling complexes to determine cellular outcomes. Caspase-8, RIPK1, and c-FLIP are central to this overlap [106,252]. DR stimulation leads to the formation of membrane-bound complex I, followed by cytosolic complex II, which can initiate apoptosis or necroptosis depending on caspase-8 activity. Active caspase-8 cleaves and inactivates RIPK1, suppressing necroptosis and favoring apoptosis. When caspase-8 is inhibited, RIPK1 and RIPK3 undergo phosphorylation and oligomerization, recruiting MLKL to form the necrosome and trigger necroptosis [45]. RIPK1 functions as a key molecular switch that is regulated by its ubiquitination status; its deubiquitination by A20 removes an inhibitory block, facilitating necrosome assembly. c-FLIP adds complexity by inhibiting apoptosis through caspase-8 blockade while modulating necroptotic signaling [252]. NFκB activation by complex I upregulates anti-apoptotic genes, promoting cell survival unless this pathway is suppressed [104,105,107,250].

Caspase-8 also bridges apoptosis and pyroptosis, beyond its traditional apoptotic role. It can directly cleave GSDMD to induce pyroptosis under certain conditions, linking apoptosis to inflammatory cell death. Caspase-1, the pyroptosis effector, can activate caspase-3 and facilitate tBid-mediated caspase-9 activation when gasdermin-dependent pyroptosis is impaired, illustrating bidirectional crosstalk between these pathways [47,267]. Inflammasomes, particularly NLRP3 upregulated by NFκB, coordinate pyroptosis by recruiting and activating procaspase-1 [266]. Overlaps between necroptosis and pyroptosis involve shared molecules like caspase-8 [251,252,267] and NFκB-mediated regulation [250,266].

**Figure 1:**
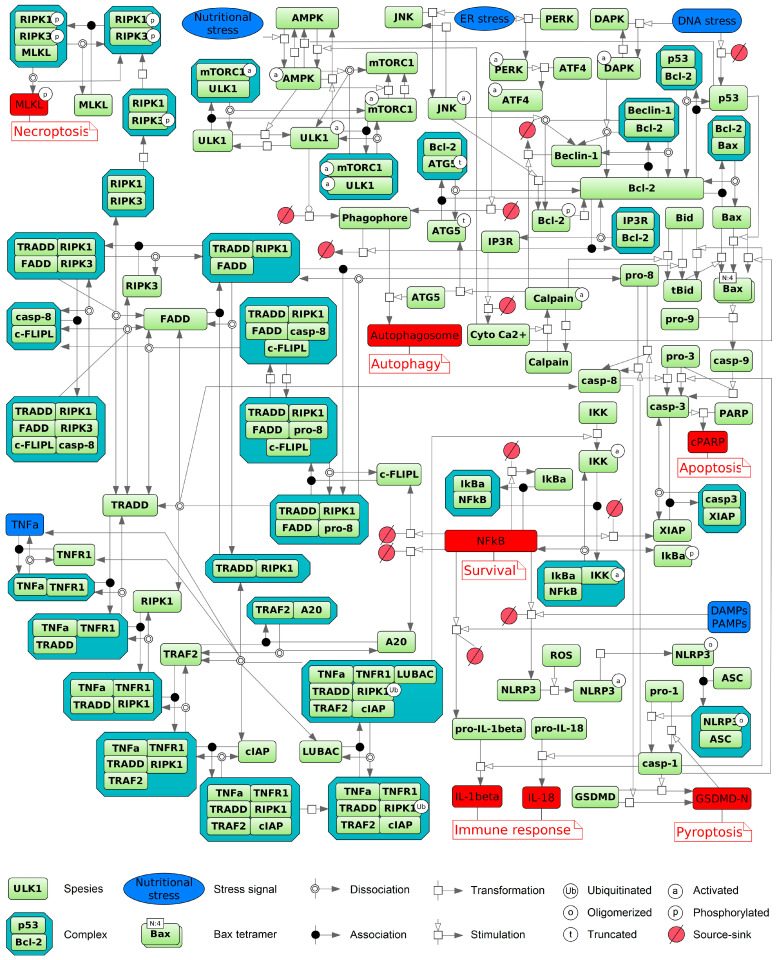
Schematic diagram of cellular signaling pathways, depicting the transduction of extracellular and intracellular input signals (blue) that culminate in diverse cell fate decisions (red), including apoptosis, autophagy, necroptosis, pyroptosis with immune response activation, and cell survival. Ferroptosis is excluded from the scheme because, at present, no ODE models directly integrate it through common components with the other represented processes. The diagram was created using BioUML software, version 2023.3 [276] and employs SBGN graphical notation [277].

We integrated these insights into a comprehensive diagram (Figure 1) summarizing key biochemical reactions across apoptosis, autophagy, necroptosis, and pyroptosis, emphasizing their roles in immune activation and cell survival. This map organizes interconnected signaling modules triggered by cellular stress, immune stimuli such as DAMPs/PAMPs, and the TNFα pathway. External signals engage membrane receptors or intracellular sensors, initiating assembly of multi-protein complexes. Upon TNFα stimulation, TNFR1 recruits TRADD, RIPK1, TRAF2, cIAPs, and the LUBAC complex to form membrane-associated complex I [105,106,252]. RIPK1 ubiquitination stabilizes this complex [252], activating the IKK complex and subsequently inducing NFκB activation [104,105,106,250]. Dominant NFκB signaling upregulates anti-apoptotic proteins such as c-FLIP, A20, and XIAP, thereby promoting cell survival [107]. Alternatively, signaling may shift to cytoplasmic complex II comprising FADD, caspase-8, and RIPK1 [105,106]. Activation of caspase-8 initiates apoptosis via executioner caspase-3, leading to PARP-1 cleavage [82,88,99,107]. In the absence of caspase-8 activity, RIPK1 and RIPK3 drive necroptosis via phosphorylation and activation of MLKL [252]. Autophagy is regulated by metabolic cues including AMPK activation and nutrient deprivation, which relieve mTORC1-mediated inhibition of ULK1 to induce autophagosome formation [44,137,139,140,148]. Pyroptosis is depicted as an inflammatory cell death driven by inflammasomes such as NLRP3. Caspase-1 activation processes pro-IL-1β and pro-IL-18 into mature cytokines and cleaves GSDMD, forming membrane pores that cause rapid cell lysis and potent immune signaling [266]. Calcium-activated calpain proteases modulate both apoptosis and autophagy [44,142]. Mitochondrial factors governed by Bax, Bcl-2, and p53 intersect these pathways [41,44,81,109,112,113,142,146,170,171]. Thus, Figure 1 illustrates a dense signaling network where the ultimate cell fate emerges from integrating survival (NFκB), metabolic (mTORC1/AMPK/ULK1), stress, and TNFR1 (TRADD/RIPK1/FADD/caspase-8) cues, with critical modulation by calcium and mitochondrial inputs. The cell’s decision among apoptosis, autophagy, necroptosis, pyroptosis, or survival reflects this intricate biochemical signaling landscape.

## 10. Concluding Remarks

Mathematical modeling is actively employed to interpret complex data, predict system behavior, and provide quantitative frameworks that complement experimental observations. The ODE-based approach, in particular, effectively models a broad spectrum of pathological and chronic conditions, including infectious diseases [278], cancer [279,280,281], heart failure [282,283], hypertension [90,91,284], chronic obstructive pulmonary disease [285], diabetes [286,287], and autoimmune disorders [288]. Therefore, this approach is indispensable for capturing the dynamic interactions within biological systems and providing insights into disease mechanisms across multiple scales.

Comprehensive ODE models of the pathways regulating cell death and survival have been developed and are continuously refined. To date, the majority of these models has focused on apoptosis, autophagy, and ICD (as a process of cell–cell interactions), while ferroptosis, pyroptosis, and necroptosis are gaining more attention (Supplementary Table S1). Notably, although this review covers autophagy in general without exploring its selective forms, there exist specific models of mitophagy–the selective degradation of damaged or dysfunctional mitochondria [138,289,290]. In contrast, no models have yet been reported for MPT-driven necrosis, entosis, NETosis, lysosome-dependent cell death, parthanatos, or for recently characterized RCD types such as alkaliptosis, oxeiptosis, cuproptosis, and disulfidptosis [9].

Beyond models of individual pathways, numerous mathematical studies capture the crosstalk between different cell death and survival mechanisms, including apoptosis and autophagy [41,43,44,141,142,146,153,156,157,158,159,161,162,164,165,166,169,170,172], apoptosis and necroptosis [45,253], as well as apoptosis and pyroptosis [46,47]. Several ICD models incorporate the role of apoptosis in modulating immune responses, addressing phenomena such as: (1) apoptosis as the predominant mode of lymphocyte death following radiotherapy [236,237,238]; (2) tumor cell expression of PD-L1 and PD-L2 promoting T cell exhaustion and apoptosis, facilitating immune evasion [208,211]; and (3) mitomycin-C chemotherapy inducing apoptosis in bladder tumor cells, which in turn activates effector immune cells [241]. Additionally, oncolytic virotherapy can induce ICD [215,220,224,232,233], with necroptosis serving as the primary underlying mechanism [253], whereas SARS-CoV-2 triggers pyroptosis [266].

Context-specific ICD modeling in cancer therapy, oncolytic virotherapy, and viral infections enhances translational insights by capturing distinct biological and immune conditions. In cancer, it enables stratification by tumor type and microenvironment, improving identification of therapeutic targets and prediction of treatment responses [207,208,229]. In oncolytic virotherapy, it improves understanding of virus–cancer cell interactions and immune modulation, guiding safer and more effective virus design and therapy regimens [215,220,224,232,233]. In viral infections, models elucidate interactions between viral replication and host immunity to inform antiviral strategies [266]. Collectively, these models improve mechanistic understanding, predict outcomes, identify biomarkers, and guide targeted interventions, thereby advancing clinical translation. Incorporating immune cell dynamics, cytokine signaling, and tumor–immune interactions into RCD models could significantly improve understanding of therapy-induced immunogenic responses by capturing the complex interplay between tumor cells and immune components. These models could clarify how immune cells such as cytotoxic T lymphocytes and dendritic cells, aided by cytokines, initiate and sustain anti-tumor immunity while revealing mechanisms of tumor resistance [291,292,293]. This enhanced insight would improve the ability to predict and optimize therapy outcomes, particularly considering the impact of non-apoptotic RCD pathways on the tumor immune microenvironment and immunotherapy efficacy [294,295]. However, comprehensive models that integrate the complex molecular events leading to regulated cell death, the release of DAMPs, subsequent intercellular immune activation, and intracellular immunogenic signaling feedback are still lacking. Applying multiscale modeling strategies—such as modular and agent-based approaches [296,297,298]—to link intracellular signaling with intercellular communication and spatial dynamics holds great potential for reproducing these complex pathways. Such approaches could enhance our capacity to predict and analyze intricate immune responses shaped by diverse cell death regulatory pathways.

In summary, although the molecular mechanisms of autophagy, apoptosis, ferroptosis, necroptosis, and pyroptosis differ significantly, these processes coexist and engage in dynamic and functional crosstalk that modulates the development of immune responses [179,299]. While a number of mathematical models have been proposed to characterize interactions between some pairs of these pathways, a comprehensive ODE model that integrates more than two modes remains to be developed. To address this gap, we present an integrated diagram of key reactions derived from the reviewed models, encompassing apoptosis, autophagy, necroptosis, pyroptosis–including its role in activating immune responses–and survival (Figure 1). This comprehensive framework establishes a foundation for follow-up quantitative modeling of the coordinated regulation of these critical cell death and survival pathways. Future modeling efforts should adopt the multi-optosis concept, which views cell death as an interconnected network rather than isolated events [300]. This approach necessitates the use of systems biology and computational modeling to integrate key signaling components, regulators, and inhibitors of apoptosis, necroptosis, pyroptosis, ferroptosis, and autophagy, alongside experimental data on pathway-specific marker activation—such as responses to chemotherapy or radiation—for rigorous calibration and validation.

Furthermore, emerging RCD types—including cuproptosis, disulfidptosis, alkaliptosis, and oxeiptosis—hold significant therapeutic potential [301,302,303,304,305,306,307,308,309,310,311,312,313]. Therefore, future research should not only refine existing programmed cell death models, which offer robust foundations and broad applicability, but also explore these novel RCD forms to uncover new mechanistic insights, particularly in oncological and metabolic vulnerabilities. This dual strategy will advance therapeutic innovation by deepening mechanistic understanding and expanding the scope of RCD modeling, thereby bridging foundational knowledge and clinical translation.

While this review focuses on ODE-based modeling of cell death and survival decisions, the complexity and multi-scale features of RCD pathways highlight the value of hybrid modeling approaches. Integrating ODEs with Boolean networks and agent-based models can better capture the full spectrum of regulatory dynamics. ODE models quantitatively describe continuous biochemical kinetics within cells, Boolean models represent logical relationships among signaling nodes and efficiently capture activation and inhibition patterns [23], while agent-based models simulate cellular behaviors and interactions within heterogeneous microenvironments [314]. Combined, these approaches provide a powerful framework that accommodates the inherent complexity of multi-omics data and dynamic immune responses.

## Figures and Tables

**Table 1:** Comparative analysis of the mathematical models discussed in this review and summarized in Supplementary Table S1.

	Number of Models	Most Used Modeling Formats			Public Availability		BioModels
		MATLAB	SBML	XPPAUT	Number of Models	Percentage of Total	
Apoptosis	40	14	12	2	20	50%	9
Autophagy	20	2	2	8	14	70%	1
ICD	46	18	13	0	17	37%	13
Ferroptosis	3	1	0	0	1	33%	0
Necroptosis	3	1	0	0	2	66%	0
Pyroptosis	2	1	0	0	1	50%	0
Apoptosis and autophagy	19	4	0	12	13	68%	0
Apoptosis and necroptosis	2	2	0	0	0	0%	0
Apoptosis and pyroptosis	2	2	0	0	1	50%	0
Total	137	45	27	22	69	50%	23

## Data Availability

Characteristics of all models discussed in this review can be accessed online via the GitLab wiki at https://gitlab.sirius-web.org/virtual-cell/info/-/wikis/Cell%20fate/Home (accessed on 11 November 2025).

## Supplementary Materials

The following supporting information can be downloaded at: https://www.mdpi.com/article/10.3390/cells14221792/s1, Table S1: Mathematical models of cell death and survival developed using ordinary differential equations.

## Abbreviations

The following abbreviations are used in this manuscript:

AMBRA1	Activating molecule in beclin-1-regulated autophagy 1
AMP	Adenosine monophosphate
AMPK	AMP-activated protein kinase
ATF4	Activating transcription factor 4
ASC	Apoptosis-associated speck-like protein containing a CARD
BH3	BCL-2 homology domain 3
BioUML	Biological universal modeling language
c-FLIPs	Cellular FADD-like interleukin (IL)-1β-converting enzyme-inhibitory proteins
CARD	Caspase recruitment domain
CHOP	C/EBP homologous protein
cIAPs	Cellular inhibitor of apoptosis proteins
CYLD	Cylindromatosis lysine 63 deubiquitinase
DAMPs	Damage-associated molecular patterns
DED	Death effector domain
DISC	Death-inducing signaling complex
DRAM	Damage-regulated autophagy modulator
DR	Death receptor
ER	Endoplasmic reticulum
GADD34	Growth arrest and DNA damage-inducible 34
GPX4	Glutathione peroxidase 4
GSDMD	Gasdermin D
GSDME	Gasdermin E
ICD	Immunogenic cell death
IκB	Inhibitor of kappa B
IKKs	IκB kinases
IL-1β, -18	Interleukin-1β, -18
iNOS	Inducible nitric oxide synthase
IRE1	Inositol requiring 1
IRF1	Interferon regulatory factor-1
FADD	Fas-associated protein with death domain
MLKL	Mixed lineage kinase domain-like
MOMP	Mitochondrial outer membrane permeabilization
MPT	Mitochondrial permeability transition
mTOR	Mammalian target of rapamycin
mTORC1	mTOR complex 1
NFκB	Nuclear factor κB
NLR	NOD-like receptor
NLRP3	NLR family pyrin domain containing 3
NO	Nitric oxide
NOD	Nucleotide-binding oligomerization domain
NRF2	Nuclear factor erythroid-related factor 2
ODEs	Ordinary differential equations
PAMPs	Pathogen-associated molecular patterns
PARP-1	Poly (ADP-ribose) polymerase-1
PD-L1, -L2	Programmed death-ligand 1, 2
PERK	Protein kinase RNA-like ER kinase
RCD	Regulated cell death
RIPK1, RIPK3	Receptor-interacting protein kinase 1, 3
RSL3	RAS-selective lethal 3
SARS-CoV-2	Severe acute respiratory syndrome coronavirus 2
SBGN	Systems biology graphical notation
SBML	Systems biology markup language
TLRs	Toll-like receptors
TNF	Tumor necrosis factor
TNFR1/2	TNF receptor 1/2
TRADD	TNFR1-associated death domain protein
TRAIL	TNF-related apoptosis-inducing ligand
TRAIL-R1/R2/R3/R4	TRAIL receptor 1/2/3/4
TRAF2	TNF receptor-associated factor 2
ULK1	Unc-51-like kinase 1
UPR	Unfolded protein response
