# Supplementary material for: Mathematical Modeling of Cell Death and Survival: Toward an Integrated Computational Framework for Multi-Decision Regulatory Dynamics
Source: Cells. 2025 Nov 14;14(22):1792. doi: 10.3390/cells14221792 (PMC12651094; doi:10.3390/cells14221792)
Supplement: Supplementary file 1 [file cells-14-01792-s001.zip › cells-3970503-supplementary.pdf]

Supplementary File

Mathematical modeling of cell death and survival: Toward an integrated computational framework for multi-decision regulatory dynamics

Elena Kutumova*, Ilya Akberdin, Inna Lavrik, Fedor Kolpakov

***Corresponding Author:** elena.kutumova@biouml.org

Table S1. Mathematical models of cell death and survival developed using ordinary differential equations.

Authors	Year	Organism	Key processes	Source of experimental data used to test or verify a model	Trigger	Program code	Open source
**Extrinsic (receptor-mediated) apoptosis**							
Albeck et al. [99]	2008	human	Subsystems of the model: 1) TRAIL-induced activation of caspase-8 by the DISC; 2) direct activation of caspase-3 by caspase-8, with caspase-3 capable of cleaving PARP except when bound to XIAP; 3) cleavage of Bid by caspase-8 to activate Bax, release of cytochrome c and Smac from mitochondria, formation of the apoptosome (which cleaves procaspase-3), and neutralization of XIAP by Smac; 4) activation of caspase-6 by caspase-3, followed by activation of caspase-8.	HeLa	TRAIL	MATLAB	+
Albeck et al. [100]	2008	human		HeLa	TRAIL	SBML, Biomodels ID: BIOMD0000000220	+
Anderson et al. [103]	2019	human	TRAIL-stimulated activation of caspase-8, followed by direct or mitochondria-mediated activation of caspase-3; AKT-induced inhibition of cytochrome c, Smac, and Bax; PTEN inhibition of AKT; and regulation of apoptosis by XIAP.	HCT116	TRAIL	MATLAB	+
Bentele et al. [82]	2004	human	CD95 DISC-mediated cleavage of procaspase-8, followed by activation of caspases-2, -3, -6, -7, and -9 (extrinsic and intrinsic pathways) and cleavage of PARP; blocking effects of c-FLIPs and IAP.	SKW 6.4	CD95L	CD95sim	+
Buchbinder et al. [94]	2018	human	Integration of two compartments: the cytosol (DISC-induced apoptotic and NFκB pathways), and the nucleus (expression of NFκB target genes).	HeLa	CD95L	SBML	+
Cho et al. [104]	2003	–	The binding of TNFα to TNFR1 with the recruitment of TRADD, which acts as a platform to recruit additional mediators: FADD, initiating the apoptosis pathway; and RIP and TRAF2, activating the proliferation pathway through NFκB.	–	TNFα	MATLAB	–
Eissing et al. [97]	2004	human	Death receptor-induced activation of caspase 8, followed by activation of caspase-3 either directly or via cleavage of Bid, release of cytochrome c and Smac from mitochondria, and cleavage of procaspase-9, including IAP-mediated inhibition.	HeLa	Receptor stimulus	MATLAB	–

Fricker et al. [87]	2010	human	Formation of procaspase-8 homo-dimers, procaspase-8/c-FLIP$_L$ dimers, and procaspase-8/ c-FLIP$_{S/R}$ dimers at the DISC.	HeLa	CD95L	SBtoolbox	+
Halder and Chatterjee [108]	2023	human	TNFα binding to TNFR2 with activation of TRAF1/2/3 and AIP1; TRAF1/2/3-induced activation of IKKs and NFκB through phosphorylation of PI3K, AKT, NIK, cIAP1/2, and RIP, promoting cell survival; and AIP1-induced activation of JNK (J), which can drive either cell survival or apoptosis.	T-regulatory cells	TNF	–	–
Hillert et al. [95]	2020	human	Caspase-8 activation in the DED filaments of the DISC, with or without FLIPinBγ (a small molecule inhibitor of c-FLIP$_L$ in the caspase-8/c-FLIP$_L$ heterodimer).	HeLa	CD95L	SBML	–
Hua et al. [83]	2005	human	Formation of the Fas DISC (1:1 noncooperative Fas-FADD and FADD-caspase-8 interactions, with the recruitment of up to three FADD and three caspase-8 molecules); DISC-induced cleavage of procaspase-8, which activates caspase-3 directly (type I cells) or via the mitochondrial pathway (type II cells); and negative regulators of apoptosis: FLIP, Bcl-2, and XIAP.	Jurkat	FasL	Entelos	–
Hua et al. [84]	2006	human	A simplified model by Hua et al. [83]: several molecules were aggregated into single surrogate molecules at various locations in the network, with the basic structure maintained.	Jurkat	FasL	Jacobian, MATLAB	–
Ivanisenko and Lavrik [96]	2020	human	Caspase-8 activation in the DED filaments of the DISC (with varying stoichiometry and composition), with or without FLIPinBγ.	HeLa	CD95L	–	–
Koh and Lee [106]	2011	–	Key modules of the model: extrinsic (TNFα-induced activation of caspase-8), intrinsic (caspase-8-triggered mitochondria-mediated activation of caspase-9), caspase (activation of caspase-3 by caspases-8 and -9), and survival (NFκB-mediated expression and synthesis of IκBα, A20, c-FLIP, and IAP).	–	TNFα	SBML	+
Kutumova et al. [88]	2013	human	Integration of the reduced models by Bentele et al. [82] and by Neumann et al. [86].	SKW 6.4, HeLa	CD95L	BioUML	+
Laussmann et al. [102]	2012	human	TRAIL-induced DISC formation; combinatorial dimerisation and trimerisation of death and decoy receptors; activation of caspase-8 mediated by c-FLIP$_L$/c-FLIP$_S$; and cleavage of an IETD FRET substrate.	HeLa	TRAIL	MATLAB	+

Mangrum and Finley [98]	2024	human	Fas-induced activation of caspase-8 and caspase-3, which are respectively inhibited by BAR and IAP proteins.	Tumor cells	FasL	MATLAB	+
Neumann et al. [86]	2010	human	CD95-mediated cleavage of procaspase-8 to p43/p41, which stimulates activation of caspase-3, and cleavage of c-FLIP$_L$ to p43-FLIP, which interacts with IKK to activate NFκB.	HeLa	CD95L	SBML, Biomodels ID: BIOMD0000000243	+
Okazaki et al. [85]	2008	human	A simplified Hua model [83]: the model was reduced as much as possible to obtain the minimal network structure showing the change in dominant pathway.	Type I/type II cells	FasL	React	–
Rangamani and Sirovich [105]	2007	–	TNFα binding to TNFR1, with the recruitment of TRADD, TRAF2, and RIP-1, followed by association with either IKK (leading to NFκB-mediated transcription of IAP and IκB) or FADD (leading to the activation of caspase-8, -3, and DNA fragmentation).	–	TNF	–	–
Schliemann et al. [107]	2011	human	TNFα-induced pro-apoptotic pathway (activation of caspases-8, -3, and -6, regulated by BAR, XIAP, and FLIP) and anti-apoptotic pathway (NFκB-mediated expression of IκBα, A20, FLIP, and XIAP mRNA).	KYM-1	TNF	SBML, Biomodels ID: BIOMD0000000407	+
Wu and Finley [93]	2017	human	TSP1-induced activation of caspase-3, followed by phosphorylation and nuclear translocation of p38MAPK; NFκB activation; and initiation of the FasL cascade.	Microvascular endothelial cells	Thrombospondin-1 (TSP1)	MATLAB	–
Zhang et al. [101]	2010	human	The model by Hua et al. [83], adapted for TRAIL signaling.	Jurkak, myeloma cells	TRAIL	MATLAB, COPASI, SBtoolbox, PottersWheel	–
**Intrinsic (mitochondria-mediated) apoptosis**							
Bagci et al. [109]	2006	mouse	Cleavage of Bid by caspase-8, leading to Bax activation and cytochrome c release from mitochondria; apoptosome formation and activation of caspases-9 and -3; inhibitory effects of IAPs and Bcl-2; cleavage of Bid and inactivation of Bcl-2 by caspase-3; and p53-mediated regulation of Bax and Bcl-2 synthesis.	JB6 C141	Silymarin	SBML, Biomodels ID: MODEL1006230056	+

Bagci et al. [111]	2008	–	Extension of the model by Bagci et al. [109] to include nitric oxide signaling pathways.	–	Extracellular stimuli, nitric oxide production	SBML, Biomodels ID: MODEL1006230064; MODEL1006230026	+
Ballweg et al. [115]	2017	–	The model incorporates three modules: p53 signaling (including the p53-Mdm2 feedback loop and Nutlin-3 inhibition of Mdm2), cisplatin signaling (activation of p53 to promote apoptosis and activation of cIAP to inhibit caspase-8, preventing apoptosis), and BH3-triggered Bax activation.	–	Cisplatin	XPPAUT, Oscill8	–
Burt et al. [122]	2022	mouse	Bax-dependent and Bax-independent regulation of caspases-3, -7, and -12 through the APRIL/NFκB and ST2/PI3K pathways, involving pro-apoptotic proteins BIM and NOXA and anti-apoptotic proteins Bcl-2 and Mcl-1.	Memory plasma cells	Mitochondrial stress	Python	–
Chen et al. [27]	2007	–	The model has three parts: 1) relocation of Bcl-2 family proteins to the mitochondrial outer membrane; 2) interactions between Bcl-2 family proteins, including Bax activation, inhibition of activated Bax and its activator via heterodimerization with Bcl-2, and displacement of the activator from Bcl-2 by activated Bax; and 3) Bax polymerization.	–	–	MATLAB	–
Hamada et al. [112]	2009	–	Extension of the Bagci model [109] incorporating DNA damage-induced p53 activation, the p53-Mdm2 feedback loop, and G2/M cell cycle arrest through inhibition of p53-mediated MPF activation by p21, 14-3-3 sigma, and GADD45.	–	High DNA damage	WinBEST-KIT	–
Legewie et al. [120]	2006	–	Apaf-1-initiated activation of caspase-9; mutual activation of caspases-3 and -9 through a positive feedback loop; and inhibition of both caspases by XIAP.	–	–	SBML, Biomodels ID: BIOMD0000000102; BIOMD0000000103	+
McKenna et al. [116]	2021	human	Extension of the model by Legewie et al. [120] to include Bax-mediated initiation of the caspase cascade and Smac-mediated sequestration of XIAP.	Melanoma cells	DNA damaging drugs	SBML, Biomodels ID: MODEL2001130002	+

Ooi and Ma [121]	2013	–	Intrinsic apoptosis model adapted from Fussenegger et al. [77] (cytochrome c binding to Apaf-1 followed by activation of caspases-9 and -3; caspase-3 inhibition by IAP), modified to include positive feedback from caspase-3 to caspase-9, an autocatalytic loop in caspase-9 activation, and mild cooperativity in caspase-3 activation.	–	–	MATLAB	+
Rehm et al. [117]	2006	human	Apoptosome-dependent activation of caspases-9, -3, and -7; regulation through inhibitory interactions with XIAP and Smac; and proteasomal degradation of all involved proteins.	HeLa	Staurosporine	SBML, Biomodels ID: BIOMD0000000256	+
Ryu et al. [118]	2008	mammals	Formation and activation of the holoenzyme complex (Apaf-1:cytochrome c:dATP:caspase-9), followed by cleavage of procaspase-3 and the fluorogenic substrate DEVD-AFC.	Extracts from 293 cells	ATP, dATP	Simpathica	–
Zhang et al. [113]	2009	–	The model has four modules: the initiator, which converts stress signals into mitochondrial Bax via BH3 proteins; the amplifier, which links Bax to cytochrome c and Smac release; the executioner, where Smac inhibits XIAP and cytochrome c activates caspase-9, leading to activation of caspase-3; and the p53 module, which covers disruption of the p53-Mdm2 loop after DNA damage, p53-induced cell cycle arrest via p21, DYRK2-driven transformation of p53 from a pro-arrest to a pro-apoptotic form, apoptosis induction by p53/p73 and enhancement by E2F1, and E2F1 inactivation by p53.	–	DNA damage	XPPAUT, Oscill8	+
**Crosstalk between extrinsic and intrinsic apoptosis**							
Fussenegger et al. [77]	2000	–	Fas-induced activation of caspase-8; stress-induced activation of caspase-9 via the Apaf-1/cytochrome c complex; activation of executioner caspases by caspase-8 and/or 9; and inhibitory effects of Bcl-xL, IAPs, and decoy proteins.	–	FasL, stress	MATLAB	–
Harrington et al. [79]	2008	human	The extrinsic, intrinsic, and coupling subnetworks with three oligomerization modules: DISC, MAC, and apoptosome.	HeLa, Jurkat T	FasL, tBid	MATLAB	–

Hendrata and Sudiono [81]	2016	human	MSC secretome pathways: FasL-mediated caspase-8 activation, DNA damage-induced p53 and caspase-9 activation, and Granzyme B- triggered caspase-10 activation (perforin pathway). All three converge on caspase-3 activation.	HeLa	Secretome derived from mesenchymal stem cells	–	–
Hong et al. [80]	2012	–	Cisplatin-induced Fas-mediated, mitochondrial (DNA damage, p53 and caspase-2 activation), and ER stress (Ca^{2+} release, procaspase-12 cleavage) pathways leading to activation of caspases-8, -9, and -3.	–	Cisplatin	MATLAB	–
Stucki and Simon [78]	2005	–	Interactions among Smac deactivators, Smac, IAPs, and caspase-3, including the mechanisms for both induction and prevention of apoptosis.	–	–	SBML, Biomodels ID: BIOMD0000001059	+
**Autophagy**							
Cook et al. [143]	2014	human	UPR and ROS signaling pathways.	LCC9	Knockdown of estrogen receptor-α, antiestrogen treatment (with fulvestrant)	–	–
Dalle Pezze et al. [129]	2016	mouse human	Effects of amino acids on the AMPK-ULK1-mTOR network.	C2C12, HeLa, MEF	Amino acids	SBML, Biomodels ID: BIOMD0000000640; MODEL1705030001; MODEL1705030000	+
Dalle Pezze et al. [138]	2021	human	Analysis of the translocation of ATG13 (part of the ULK complex) to autophagic puncta during starvation-induced autophagy and ivermectin-induced mitophagy with wortmannin treatment.	HEK293	Starvation (autophagy induction), ivermectin (mitophagy induction)	SBML	+
Deter [126]	1975	rat	Telolysosome-autophagosome-autolysosome interaction.	Liver cells	Glucagon	–	–
Hajdú et al. [151]	2022	human	Dynamic characteristics of the mTORC1-ULK1-PP2A regulatory triangle.	HEK293T	Rapamycin, okadaic acid	XPPAUT	+
Hajdú et al. [152]	2023	human	The AMPK-ULK1-mTORC1 regulatory triangle, controlled by an extra regulatory protein.	–	Cellular stress, mTOR inhibition by rapamycin	XPPAUT	+

Han et al. [127]	2015	mammals	A three-component description of the autophagy process, including proteins/organelles, autophagosomes, and autolysosomes.	Hepatocytes	–	–	–
Han et al. [128]	2020	human	A four-component description of the autophagy process, including intracellular proteins, autophagosomes, autolysosomes, and extracellular amyloid-β peptides.	Neurons	Abnormal increase in amyloid-β peptide formation in Alzheimer's disease	–	–
Holczer et al. [147]	2019	human	Dynamic characteristics of the AMPK-ULK1-mTORC1 regulatory triangle.	HEK293T	Rapamycin, starvation	XPPAUT	+
Holczer et al. [149]	2020	human	Dynamic characteristics of the AMPK-ULK1-mTORC1 regulatory triangle.	HEK293T	Rapamycin, starvation	XPPAUT	+
Kapuy et al. [155]	2018	–	Dynamic characteristics of the AMPK-NRF2-mTOR regulatory triangle.	–	Oxidative stress	XPPAUT	+
Kapuy et al. [150]	2021	–	Key model elements include an autophagy inducer (AMPK, GADD34, etc.), an autophagy controller (ULK1/2, CHOP, NRF2, etc.), mTORC1, and an autophagy executor (Beclin-1, etc.).	–	Stress signals (starvation, ER stress, oxidative stress)	XPPAUT	+
Kapuy et al. [160]	2021	human mouse rat	KRAS and mTOR pathways.	KRAS mutant cancer cells	KRAS pathway	XPPAUT	+
Kapuy et al. [154]	2024	human mouse monkey rat	Dynamic characteristics of the AMPK-ULK1-mTORC1 regulatory triangle.	HEK293FT, HEK293 c18, 3T3-L1, U2OS, MEF, murine liver and primary hepatocytes, HEK293T, COS7, C2C12, murine satellite cells, HeLa, HT-29, HCT116, HepG2, A549, HT22, SU86.86, Panc04.03, HEK293A, 4T1, SKOV3, OVCAR, B16-F0, A431, 661 W, C57BL/6, NRK-52E, NIH3T3, ARPE-19, CF7, schizosaccharomyces pombe, *in vivo* experiments	Stress signals	XPPAUT	–
Pavel et al. [173]	2021	human mouse	Dynamics of autophagy levels, YAP/TAZ activity, and α-catenin levels in the context of autophagy impairment.	MCF10A, HEK293T, HeLa, pMECs, pMEFs, HepG2, THLE2, A549, Huh7, MCF7, primary mouse cortical neurons	EBSS-induced starvation, Tat-Beclin1, Trehalose, SMER28	MAPLE	+

Sadria and Layton [139]	2021	mouse	The model includes the insulin/IGF-1 pathway; amino acid sensors and mTORC1 regulation; the Preiss-Handler and salvage pathways regulating NAD+ and SIRT1 metabolism; the energy sensor AMPK; and the transcription factors FOXO and PGC-1α.	C2C12 and limited data from mouse kidney cells, rat cells, human cells	AMPK activation	MATLAB	+
Sadria et al. [140]	2022	human mouse	The model incorporates key proteins involved in cellular metabolism (IRS and AKT), the mTOR pathway (mTORC1, mTORC2, DEPTOR, and ULK1), and key energy sensors (AMPK and SIRT1).	–	AMPK activation	MATLAB	+
Sarmah et al. [171]	2021	human mouse	A seven-dimensional model connecting p53, DNA damage, and autophagy in lung cancer, featuring a circuit with p53, Mdm2, MdmX, AMPK, mTOR, Bcl-2, and Beclin-1.	NIH 3T3, MCF7	p53 induction following DNA damage	–	–
Shirin et al. [137]	2019	human	Regulatory interactions among mTORC1, ULK1, AMPK, and VPS34, as well as the pharmacokinetics of inhibitors specific to these kinases.	U2OS	Drugs (rapamycin, buparlisib, SBI-206965, dorsomorphin, PF-06409577, SAR405)	PSOPT	+
Szymańska et al. [148]	2015	human	Behavior of the AMPK-ULK1-mTORC1 network.	–	Rapamycin, intrinsic AMPK kinase activity	BioNetGen	+
**Ferroptosis**							
Arbatskiy et al. [183]	2024	–	The model comprises seven modules: 1) Fenton's reaction, involving the oxidation of iron ions from Fe^{2+} to Fe^{3+}; 2) iron metabolism, covering iron transport across the cell membrane and its translocation into mitochondria and lysosomes; 3) lipid synthesis, driven by ACSL4 and LPCAT3, which esterify PUFAs into PE; 4) lipid peroxidation, describing the LOX-initiated sequential transformation of PE-PUFAs into phospholipid hydroperoxides; 5) the pentose phosphate pathway, an alternative glucose oxidation route producing NADPH and ribose-5-phosphate; 6) the antioxidant system, where GPX4 reduces hydrogen peroxide, hydroperoxides, and lipid peroxides by oxidizing GSH to GSSG; and 7) GSH synthesis via the gamma-glutamyl cycle.	–	Peroxidation of polyunsaturated lipids	BioUML	–

Kagan et al. [181]	2017	mouse	The model has three modules: 1) Arachidonoyl (AA) metabolism: PLA2 catalyzes the release of AA from membrane phospholipids; AA is oxidized by 5/12/15-LOX into 5/12/15-HPETE, which GPX4 reduces to 5/12/15-HETE using GSH; 5-LOX converts 5-HETE into LTA4; AA is oxidized by COX to PGH2, producing PGE2; feedback occurs through 5/12/15-HPETE, 5/12/15-HETE, LTA4, and PGE2. 2) AA induced ferroptosis: Acsl4 catalyzes the formation of AA-CoA, which elongase converts to AdA-CoA; AA-CoA and AdA-CoA are either broken down or esterified into PE as PE-AA and PE-AdA by Lcat3, then converted by LOXes to PE-AA-OOH and PE-AdA-OOH, whose accumulation triggers ferroptosis. 3) GPX4 dependent regulation: System xc- imports Cys and exports Glu; GCS and GS synthesize GSH from Cys, Glu, and Gly; GPX4 uses GSH to reduce PE-AA-OOH and PE-AdA-OOH to PE-AA-OH and PE-AdA-OH, producing GSSG; GR regenerates GSH from GSSG.	MEF, MLE	RSL3	COPASI	–
Kapralov et al. [182]	2020	mouse human	ASCL4-, LPCAT3-, and 15-LOX-stimulated production of HOO-AA-PE and its cleaved forms—oxidatively truncated species (PE_{OxTr}), whose accumulation leads to ferroptosis; reduction of HOO-AA-PE into HO-AA-PE by GPX4 while converting GSH to GSSG; and iNOS- and NO• donors-induced production of NO•, which inactivates 15-LOX and reacts with HOO-AA-PE and PE_{OxTr} to form nitroxygenated lipid products.	RAW 264.7, bone marrow cells, EOC 20, primary mouse microglial cells, MLE, HBE, *in vivo* experiments	RSL3, supernatants obtained from *Pseudomonas aeruginosa*	BioNetGen , C, MATLAB	+
**Immunogenic cell death**							
Adam [205]	1996	mammals	Adaptation of the model by DeLisi and Rescigno [193] to include the effects of vascularization within a tumor or multicellular spheroid.	Solid tumor	Anti-tumor immune response (via reactive lymphocytes)	–	–
Alfonso et al. [237]	2020	mouse	Extension of the model by Hatzikirou et al. [206] to account for the effects of radiotherapy on antitumor immune responses.	Colon carcinoma cells, CT26	Radiotherapy	MATLAB	–

Bunimovich-Mendrazitsky et al. [229]	2007	human	Interactions among tumor cells (infected and uninfected), effector cells, and Bacillus Calmette–Guérin.	Superficial bladder cancer	Continuous immunotherapy with Bacillus Calmette–Guérin	SBML, Biomodels ID: BIOMD0000001034	+
Byun et al. [214]	2020	mouse	Interactions among tumor cells, $CD8^+$ T cells, and PD-L1/PD-1, influenced by radiotherapy and immune checkpoint blockade.	TUBO cells from a spontaneous mammary tumor	Anti-PD-L1, ionizing irradiation therapy	MATLAB, Berkeley Madonna	–
de Boer and Hogeweg [198]	1985	–	A series of models on cytotoxic T-cell activation, derived by successive simplifications from the model for tumor escape from immune elimination by Grossman and Berke [197].	Tumor cells	Anti-tumor immune response (via cytotoxic T cells)	GRIND, DEASIM	–
de Boer and Hogeweg [200]	1986	mouse	The model by de Boer et al. [199], in which only one parameter is changed (i.e., effector longevity).	SL2 tumor	Anti-tumor immune response (via cytotoxic macrophages and T cells)	GRIND, SBML, Biomodels ID: MODEL1912110001	+
de Boer et al. [199]	1985	mouse	The model specifies the induction of cytotoxic T cells and antigen presentation by macrophages, which activates helper T cells, as well as the production of lymphoid factors that induce cytotoxic macrophages, T cell proliferation, and inflammation. Tumor escape mechanisms (suppression, antigenic heterogeneity) are omitted.	Ascitic SL2 tumor	Anti-tumor immune response (via cytotoxic macrophages and T cells)	GRIND, SBML, Biomodels ID: MODEL1911130003	+
de Pillis et al. [204]	2005	mouse human	Interactions among tumor cells, NK cells, and tumor-specific $CD8^+$ T cells involving growth, death, killing, recruitment, and inactivation.	Tumor cells	Anti-tumor immune response (via natural killer and $CD8^+$ T cells)	MATLAB, SBML, Biomodels ID: MODEL1907260001	+
de Pillis et al. [222]	2006	mouse human	Interactions among tumor cells, NK cells, $CD8^+$ T cells, and circulating lymphocytes, considering responses to chemotherapy, immunotherapy (interleukin-2 and tumor-infiltrating lymphocyte injections), and vaccine therapy.	Tumor cells	Immune, vaccine and chemotherapy treatments	–	–
DeLisi and Rescigno [193]	1977	–	Predator-prey model involving specifically reactive lymphocytes stimulated by, and antagonistic to, the tumor.	Solid tumor	Anti-tumor immune response (via reactive lymphocytes)	–	–
Eftimie et al. [224]	2011	mouse	Interactions among central memory and effector $CD8^+$ T cells, tumor cells (uninfected and infected), vesicular stomatitis virus, and adenovirus across lymphoid (immune activation) and peripheral (tumor site) tissue compartments.	Aggressive intracranial B16 melanoma	Oncolytic virus (vesicular stomatitis virus), vaccine virus (adenovirus)	–	–

Forjanič and Miklavčič [240]	2017	mouse	Tumor-immune interactions under chemotherapy or electrochemotherapy with bleomycin or cisplatin.	Subcutaneous SA-1 sarcoma tumors, LPB sarcoma	Bleomycin or cisplatin alone or combined with high-voltage electric pulses (in SA-1 sarcoma mice); chemotherapy and electrochemotherapy with varying cisplatin doses (in LPB sarcoma mice)	MATLAB	–
Grossman and Berke [197]	1980	–	Interactions among lymphocytes, tumor cells, and blocking factors: 1) tumor-stimulated precursor T cells proliferate and differentiate into killer cells that destroy tumor cells; 2) tumor-shed antigens act as blocking factors that interfere with the immune response.	Tumor cells	Anti-tumor immune response (via cytotoxic T cells)	–	–
Hatzikirou et al. [206]	2015	mouse	A model of tumor-effector cell interactions that incorporates the influence of tumor vasculature on tumor growth and immune response dynamics.	CT26	Anti-tumor immune response	MATCONT	–
Kim et al. [218]	2023	human	Extension of the model by Sung et al. [239] to account for the effects of anti-CTLA-4 immune checkpoint inhibitors.	Hepatocellular carcinoma	Radiotherapy and CTLA-4 immune checkpoint inhibition (tremelimumab)	Python	–
Kirschner and Panetta [226]	1998	–	Interactions among tumor cells, immune-effector cells, and the cytokine interleukin-2.	Tumor cells	Immunotherapy treatment	SBML, Biomodels ID: BIOMD0000000732	+
Kirshtein et al. [207]	2020	human	Interactions among key components of the immune microenvironment in colon cancer: cancer cells, naive T cells, helper T cells, cytotoxic cells ($CD8^+$ T cells and NK cells), regulatory T cells, naive dendritic cells, activated dendritic cells (antigen-presenting cells), macrophages, necrotic cells, HMGB1, carcinogenic cytokines (IL-6, IL-17, IL-21, and IL-22), immunosuppressive agents (IL-10 and CCL20), IFN-γ, and TGF-β.	Colon cancer cells	Anti-tumor immune response (via cytotoxic T-lymphocytes and possibly NK cells)	Python, MATLAB	+

Kosinsky et al. [211]	2018	mouse	QSP model of the cancer immunity cycle incorporating radiotherapy and PD-L1 blockade: radiation-induced tumor cell death promotes dendritic cell maturation and tumor antigen buildup, driving tumor-infiltrating T cell recruitment and PD-L1 upregulation; increased tumor antigens foster accumulation of immunosuppressive cells, including regulatory T cells, which—together with PD-L1—regulate immune activation; and the tumor-killing effects of radiotherapy, as well as the PK/PD of anti-PD-1/L1 mAbs, are also included.	CT26	Radiation and anti-PD-(L)1 therapies	IQM toolbox (based on MATLAB), R, Monolix, SBML, Biomodels ID: BIOMD0000000863	+
Kuznetsov and Knott [209]	2001	mouse	Deterministic models of tumor growth, suppression, and regrowth that include adjuvant immunotherapy through increased killer cell infiltration or proliferation in dormant cancers.	BCL_1 lymphoma in the spleen	Immune attack of cytotoxic killer cells	MLAB	–
Kuznetsov et al. [201]	1994	mouse	A model of the cytotoxic T lymphocyte response to immunogenic tumor progression, including immunostimulation of tumor growth, tumor sneaking through, and tumor dormancy.	BCL_1 lymphoma in the spleen	Immune attack by cytotoxic effector cells, e.g. CTL or NK cells	SBML, Biomodels ID: BIOMD0000000762	+
Leon et al. [223]	2007	–	The model includes two compartments (tumor site and adjacent lymph node) with cell migration, capturing interactions among regulatory T cells, effector T cells, antigen-presenting cells, and tumor cells in response to vaccination, regulatory T cell suppression, and incomplete surgery.	Solid tumor	Vaccination, immune suppression, surgery, or combinations of these therapies	Mathematica	–
López Alfonso et al. [236]	2019	human	Extension of the model by Kuznetsov and Knott [209] to explore tumor-effector T cell interactions under radiotherapy and surgery.	Tumor cells	Surgery followed by radiotherapy, radiotherapy before surgery, and radiotherapy alone	MATLAB	–
Montaseri et al. [238]	2020	mouse	Extension of the model by Hatzikirou et al. [206] to include immunological effects of radiotherapy.	CT26	Radiotherapy	MATLAB	–
Moore and Li [203]	2004	human	Interactions among naive T cells, effector T cells, and chronic myelogenous leukemia (CML) cells.	CML cells	Immune response to CML (via effector T cells)	Mathematica, SBML, Biomodels ID: BIOMD0000000733; BIOMD0000000662	+

Nikolopoulou et al. [213]	2018	mouse human	Tumour-immune interactions after T cell activation: tumour cells express PD-L1; activated T cells express PD-1 and PD-L1; activated T cells kill tumour cells unless the PD-1–PD-L1 complex forms, which inhibits T cell function; anti-PD-1 blocks PD-1, preventing this inhibition.	Tumor cells	Immune checkpoint inhibition (anti-PD-1): continuous drug *vs.* periodic injections	MATLAB, SBML, Biomodels ID: MODEL1908270001	+
Okuneye et al. [216]	2021	mouse	A model of FGFR3-driven bladder cancer growth (including the local evolution of free FGFR3 monomer receptors and active FGFR3 dimer complexes on tumor cells, as well as PD-1- and PD-L1-mediated cytotoxic T cell killing) under combined treatment with a FGFR3 inhibitor and a PD-1/PD-L1-targeting monoclonal antibody.	MB49	Anti-PD-L1 therapy, anti-FGFR3 therapy (with rogaratinib)	MATLAB	–
Poleszczuk and Enderling [212]	2018	mouse	A model of radiation response, radiation-induced immune activation, checkpoint blockade, and inter-exchange of activated T cells between two tumor sites, where they may extravasate with a certain time-dependent probability, promoting cancer cell death.	Breast carcinoma cells	Radiation and/or concurrent systemic immunotherapy (immune checkpoint blockade with anti-CTLA-4 antibody)	MATLAB	–
Poleszczuk et al. [210]	2016	human	The model describes the trafficking of radiotherapy-activated cytotoxic T cells through four compartments (lungs; liver; gastro-intestinal tract and spleen; and other systemic organs), where they infiltrate N distinct tumors in the lungs, liver, breast, or kidneys with a certain probability.	Metastatic tumors in the lungs, liver, breast, and kidneys	Radiotherapy applied to one of the tumors	MATLAB	–
Rescigno and DeLisi [194]	1977	–	Improvement of the model by DeLisi and Rescigno [193] by introducing a delay in the formation of killer lymphocytes.	Tumor cells (*de novo* development)	Anti-tumor immune response (via killer lymphocytes)	–	–
Robertson-Tessi et al. [225]	2012	human	Interactions among tumor cells, dendritic cells, $CD8^+$ effector T cells, $CD4^+$ helper T cells, $CD4^+CD25^+Foxp3^+$ regulatory T cells, the proliferative cytokine IL-2, and the suppressive cytokines IL-10 and TGF-β.	Tumor cells	Anti-tumor immune response (via effector T cells), dendritic cell therapy	MATLAB, SBML, Biomodels ID: BIOMD0000000731	+
Rodrigues et al. [227]	2019	human	Interactions between neoplastic B-lymphocytes and “healthy” T-lymphocytes under chemotherapy and immunotherapy.	Neoplastic B-lymphocytes (chronic lymphocytic leukemia)	Chemotherapy and adoptive cellular immunotherapy	SBML, Biomodels ID: BIOMD0000000879	+
Rodríguez-Pérez et al. [230]	2007	–	The model by Sotolongo-Costa et al. [228], modified to include a time delay in immune system stimulation through its interaction with the tumor.	Tumor cells	Cytokine-based periodic immunotherapy treatment	–	–

Senekal et al. [233]	2021	–	Interactions among tumor cells (infected and uninfected), an oncolytic virus, and NK cells.	Tumor cells	Oncolytic virotherapy, NK cell therapy	MATLAB	–
Shaikhet and Bunimovich-Mendrazitsky [231]	2018	human	Refinement of the model by Bunimovich-Mendrazitsky et al. [229] to incorporate cytotoxic T lymphocyte differentiation as an integral element of the delayed immune response to Bacillus Calmette-Guérin therapy, along with logistic growth terms for cancer cell proliferation.	Bladder cancer	Immune response to Bacillus Calmette-Guérin	–	–
Sofia et al. [208]	2022	human	Interactions among key elements of the clear cell renal cell carcinoma microenvironment: cancer cells, helper T cells (activated memory CD4 T cells, follicular helper T cells), cytotoxic cells (CD8 T cells, activated NK cells), regulatory T cells, naive T cells (naive CD4 T cells, memory resting CD4 T cells, resting NK cells), naive dendritic cells, mature dendritic cells, naive macrophages (M0, monocytes), macrophages (M1 and M2), necrotic cells, IFN-γ, HMGB1, IL-10, IL-2/IL-12, and IL-6.	Clear cell renal cell carcinoma	Anti-tumor immune response (via $CD8^{+}$ T cells and NK cells)	Python, MATLAB	+
Sotolongo-Costa et al. [228]	2003	–	Predator–prey model with lymphocyte diffusion, tumor aggressiveness, and cytokine impact.	Tumor cells	Periodical immunotherapy treatment with cytokines	SBML, Biomodels ID: BIOMD0000000785	+
Sotolongo-Grau et al. [234]	2009	human	The model by Sotolongo-Costa et al. [228], modified to include the effects of radiotherapy.	Tumor cells	Radiation therapy	–	–
Storey et al. [215]	2020	mouse	Glioblastoma treatment model: the oncolytic virus lyses susceptible tumor cells, releasing new viral particles; innate immune cells (macrophages, NK cells) activate upon encountering viral particles or infected cells, then target and kill them, and also present viral/ tumor antigens to $CD8^{+}$ T cells, activating antiviral and antitumor T cells; antiviral T cells kill infected cells and viral particles; antitumor T cells kill both infected and susceptible tumor cells; adaptive immune cell activity is suppressed via the PD-1/PD-L1 checkpoint.	Glioblastoma	Oncolytic viral therapy (with herpes simplex virus), immune checkpoint (PD-1/PD-L1) inhibition (with nivolumab)	–	–
Sung et al. [239]	2020	human	Interactions among tumor cells (primary, inactivated antigen-releasing, and metastatic) and circulating lymphocytes, with radiation targeting the primary tumor and circulating lymphocytes.	Hepatocellular carcinoma	Radiation	–	–

Sung et al. [217]	2022	human	Extension of the model by Sung et al. [239] to account for increased effector lymphocyte-mediated cell killing induced by immune checkpoint inhibition.	Hepatocellular carcinoma	Immune checkpoint (PD-L1) inhibitors (durvalumab) combined with radiation therapy	–	–
Walker et al. [235]	2018	mouse	Adaptation and integration of models by Kuznetsov and Knott [209] and Poleszczuk et al. [210] to study tumor-immune cell interactions at each metastatic cancer site, both with and without therapy (surgery, radiation).	BCL_1 lymphoma in the spleen	Anti-tumor immune response (via cytotoxic T cells), radiotherapy, surgery	MATLAB	–
Wang et al. [219]	2023	mouse	Interactions between high- and low-antigen tumor cells and cytotoxic T cells, involving the PD-1/PD-L1 checkpoint: T cells kill tumor cells via fast perforin/granzyme and slow FasL pathways; PD-1 on T cells binds to PD-L1 on tumor cells to inhibit T cell activity; anti-PD-1 blocks this binding; cytokine therapy and adoptive T cell transfer are simulated by altering model parameters.	Tumor cells	Immune checkpoint (PD-1/PD-L1) inhibition, cytokine therapy, adoptive T cell transfer	–	–
Wodarz [232]	2001	–	Interactions among tumor cells, the tumor-infecting virus, and virus-specific cytotoxic T-lymphocytes (CTLs), considering three scenarios: (a) viral cytotoxicity alone kills tumor cells; (b) a virus-specific CTL response contributes to the killing of infected tumor cells; and (c) the virus stimulates a tumor-specific CTL response.	Tumor cells	Tumor-specific viruses (e.g., adenovirus ONYX-015)	SBML, Biomodels ID: BIOMD0000001043	+
Yao et al. [221]	2024	human	Interactions among sensitive and resistant tumor cells and $CD8^+$ T cells under immune checkpoint inhibitor and chemotherapy treatments.	Tumor cells	Immune checkpoint inhibitor therapy combined with chemotherapy	Python	+
Yosef and Bunimovich-Mendrazitsky [241]	2024	human	A model of bladder cancer eradication after mitomycin-C treatment: tumor cells proliferate while effector cells are steadily produced; mitomycin-C acts directly by inhibiting DNA synthesis, which causes tumor cell cycle arrest and apoptosis, and indirectly by activating effector cells through apoptotic tumor cells to eliminate the latter.	Non-muscle-invasive bladder cancer	Mitomycin-C chemotherapy	MATLAB	+

Yu et al. [220]	2023	mouse	A model of malignant melanoma B16-CD20 treatment in mice with oncolytic virus therapy and anti-CTLA-4: susceptible tumor cells are infected and lysed, releasing new viral particles; fragments from infected cells stimulate antiviral immune cells to kill infected cells and clear free virus; $CD4^+$ and $CD8^+$ T cells, along with NK cells, attack both infected and susceptible tumor cells; NK cells activate upon encountering the virus and recruit T cells; IFN-γ, upregulated by susceptible and infected tumor cells, $CD4^+/CD8^+$ T cells, and NK cells, promotes tumor cell death; CTLA-4, expressed by $CD4^+/CD8^+$ T cells, suppresses $CD4^+$ T cells and IFN-γ; NK cells, activated by ICD of infected tumor cells and IFN-γ, clear free virus.	Malignant melanoma B16-CD20 cells	Combined therapy of oncolytic viruses and checkpoint inhibition (with anti-CTLA-4)	MATLAB	–
**Necroptosis**							
Ildefonso et al. [252]	2023	mouse	Sequential formation of complex I (TNF-TNFR1- TRADD-ubiquitinated RIPK1-TRAF2-cIAP1/2- LUBAC) and complex II (deubiquitinated RIPK1-TRADD-FADD), which is then modified into complex IIa (complex II-procaspase-8-c-$FLIP_L$), leading to necroptosis inhibition, or into complex IIb (complex II-RIPK3), resulting in the formation of the necrosome (pRIPK1-pRIPK3-MLKL), release of pMLKL, and necroptosis.	L929	TNF	PySB	+
Oliver Metzig et al. [250]	2020	mouse	The model consists of three modules: the TNFR-IKK and IκB-NFκB modules, which depict TNF-induced formation of complex I that activates IKK, leading to degradation of IκB proteins and stimulation of NFκB to initiate transcription of IκBs and A20 (with A20 counteracting complex I activation); and a necroptosis module, where complex I activates RIPK1, RIPK3, and pMLKL, with A20 inhibiting RIPK3.	L929	TNF	MATLAB	+
Xu et al. [251]	2021	–	TRADD-RIPK1-caspase-8-RIPK3 circuit.	–	TNF	–	–

**Pyroptosis**							
Hamis and Macfarlane [266]	2021	human	A single-cell pyroptosis model in SARS-CoV-2 infection: when TLRs detect viral PAMPs, NFκB translocates to the nucleus and triggers the synthesis of NLRP3 and pro-IL-1β; once activated, NLRP3 oligomerizes and recruits ASC and procaspase-1; activated caspase-1 cleaves GSDMD, pro-IL-1β, and pro-IL-18; the cell ruptures when its volume reaches a critical threshold due to swelling; and drug action is modeled as binding to active NLRP3.	Epithelial cells, immune cells	SARS-CoV-2	MATLAB	+
Zhu et al. [267]	2022	mouse	A coarse-grained model of pyroptosis and secondary pyroptosis, including seven key constituents: caspase-1, caspase-3, caspase-8, caspase-9, tBid, GSDMD, and GSDME.	Bone marrow-derived macrophages	–	Python	–
**Crosstalt between autophagy and apoptosis**							
Ge and Wang [170]	2020	mammals	The choice between autophagy and apoptosis regulated by Ca^{2+}, mTOR, Bax, Bcl-2, Beclin-1, and caspases.	–	ER stress, nutritional stress	MATLAB, XPPAUT	–
Hajdú et al. [169]	2024	human<br>rat<br>E.coli<br>eukaryote	Reproduction and calibration of the model by Liu et al. [44].	HeLa, MCF7, E.coli cells, eukaryotic cells, rat kidney proximal tubular cells, NSCLC cells, SB2 melanoma cells	Stress signals	Optima++	+
Holczer et al. [162]	2015	human	The components involved in the ER stress response are grouped into three modules: ER stress sensor, autophagy inducer, and apoptosis inducer.	HepG2, HEK293	ER stress (thapsigargin, tunicamycin, dithiothreitol)	XPPAUT	+
Holczer et al. [153]	2024	human	ER stress-response mechanism that includes the following elements: ER stress sensor, autophagy inducer, mTORC1, autophagy effector, and apoptosis effector.	HEK293T	Sulforaphane	XPPAUT	+
Kapuy and Korcsmáros [159]	2022	human	The key elements of the model include two active forms of the ER stress sensor (one for autophagy-dependent survival and one for apoptosis), an autophagy inducer, an autophagy controller, and an active autophagosome.	–	ER stress	XPPAUT	+

Kapuy et al. [146]	2013	mouse human	Interactions between Bax, Bcl-2, Beclin-1, and caspases.	Ba/F3, HeLa	Stress signals (interleukin-3 depletion, starvation, staurosporine, tunicamycin, thapsigargin, rapamycin, camptothecin)	XPPAUT	+
Kapuy et al. [161]	2014	human	Interplay of mTOR and UPR (the unfolded protein response) in the control of cell survival and cell death decisions.	HepG2, HEK293	ER stress (thapsigargin, tunicamycin), mTOR inhibitors (rapamycin, metyrapone)	XPPAUT	+
Kapuy et al. [157]	2020	human mouse rat monkey hamster	GADD34-CHOP connections within the PERK branch of the unfolded protein response.	HCT116, HEK293T, MEF, COS-1, 293T, CHO K1, HEK, INS-1, mouse tissues, ML-1, WMN, Ovcar-4, A549, RKO, Hep1-6, HepG2, Huh7, Hep3B, A94, Rat1, Rat1-Myc, Neo, Huh-7, MIN6, human and murine AML cells, THP-1, human umbilical vein endothelial cells, HEI-OC1, bone narrow-derived macrophage, HeLa, MCF7.beclin1, MCF7, HT-29, Jurkat, E16.5, *in vivo* experiments	ER stress	XPPAUT	+
Liu et al. [44]	2017	human	mTOR and inositol autophagy pathways, intrinsic apoptosis pathways, and their crosstalks mediated by Bc-l2, caspases, p53, calpain and Ca^{2+}.	Neuroglioma H4 cells	Stress signals (staurosporine, tunicamycin, Torin 1)	SUNDAILS package	–
Márton et al. [156]	2017	human	The key elements of the model include an autophagy inducer, an apoptosis inducer, and the ER stress sensors PERK and IRE1.	HEK293T	ER stress (thapsigargin, tunicamycin)	XPPAUT	+
Márton et al. [158]	2022	human	PERK-induced ATF4-GADD34-CHOP regulatory triangle.	HEK293T	ER stress (thapsigargin)	XPPAUT	+
Mehta et al. [141]	2022	mouse human	QSP framework including host immune response, AMPK-mTOR signaling, and PK/PD models for metformin and antibiotics against *M. tuberculosis.*	M. tuberculosis infected macrophages	Metformin in combination with antibiotics	R	+
Parmar et al. [43]	2013	human	The choice between autophagy and apoptosis, regulated by UPR, Ca^{2+}, mTORC1, Bax, Bcl-2, Beclin-1, and NFκB.	MCF7/LCC9	ICI 182780	MATLAB	+

Schwartz-Roberts et al. [172]	2015	human	A model of antiestrogen-sensitive MCF7/LCC1 cells, incorporating the key molecular components (estrogen receptor-α, IRF1, and ATG7) and their interactions.	MCF7, T47D, BT-474, MDA-MB-231, MCF7/LCC1, MCF7/LCC9	ICI 182780	MATLAB	–
Tavassoly et al. [142]	2015	rat	JNK/Beclin-1, DAPK/Beclin-1, and BH3 pathways.	Renal proximal tubular cells	Stress signals (cisplatin)	MATLAB	+
Tyson et al. [41]	2011	human	IRE1/JNK/Beclin-1, and DAPK/Beclin-1 pathways.	MCF7	Stress signals	XPPAUT	+
Yang and Yang [165]	2024	–	A three-component model of stress-induced, Beclin-1-dependent autophagy and caspase-mediated apoptosis with double negative feedback loops between Beclin-1 and caspases.	–	Stress signals	XPPAUT, Matcont	–
Yang et al. [164]	2019	–	Interactions between Bax, Bcl-2, AMBRA1, Beclin-1, and caspases.	–	Stress signals (transient nutrient starvation, DNA damage or growth factor withdrawal)	–	–
Yang et al. [166]	2023	human	Interactions between ROS, misfolded α-synuclein, ER stress, mTOR, Beclin-1, and caspases.	Neurons	Stress signals (oxidative stress such as nutrient starvation, mitochondria dysfunction, and the loss of dopamine; reduction in the age-related anti-oxidative mechanism; genetic damage that promotes the formation of misfolded α-synuclein)	XPPAUT	–
**Crosstalt between necroptosis and apoptosis**							
Lee et al. [253]	2024	–	A regulatory network in the tumor microenvironment influenced by oncolytic virus–bortezomib therapy that disrupts intracellular signaling pathways (IκB, NFκB, Bax, RIPK1), leading to apoptosis or necroptosis.	–	Bortezomib and oncolytic virotherapy	MATLAB	–

Li et al. [45]	2021	mouse	TNF signaling network, including complex I (TRADD and RIPK1 competitively binding to TNFR1), complex II (RIPK1, FADD, and caspase-8), and the necrosome (complex II plus RIPK3). In the necrosome, caspase-8 inactivates RIPK1 and RIPK3 to inhibit necroptosis, while uncleaved RIPK3 phosphorylates and recruits MLKL to trigger it. Activation of procaspase-8 occurs via three mechanisms: through TRADD, RIPK1, or both.	L929	TNF	MATLAB, Python	–
**Crosstalk between pyroptosis and apoptosis**							
Li et al. [47]	2022	mouse	Interplay between NLRP1b, caspases-1, -3, -8, and -9, ASC, and GSDMD.	RAW 264.7, J774A.1	Lethal toxin	MATLAB	+
Yin et al. [46]	2021	mouse	A coarse-grained model of pyroptosis and apoptosis, including five key constituents: caspase-1, caspase-3, caspase-8, caspase-9, and GSDMD.	RAW264.7, Colon-26	Salmonella Typhimurium infection	MATLAB	–
